# Burden of ischemic heart disease in the Middle East and North Africa (MENA) and attributable risk factors: An epidemiological analysis from 1990 to 2019

**DOI:** 10.1016/j.ijcha.2023.101316

**Published:** 2023-12-03

**Authors:** Mohammad Ahmadi, Shana Ahadi, Mohammad Amin Khadembashiri, Mohamad Mehdi Khadembashiri, Mehrdad Mahalleh, Hani AziziKia, Hamid Reza Zare, Amir Sobh Rakhshan Khah, Hamidreza Hekmat, Rajabali Daroudi, Ali Akbari Sari

**Affiliations:** a Students' Scientific Research Center, Tehran University of Medical Sciences, Tehran, Iran; b School of Medicine, Jondishapour University of Medical Sciences, Ahvaz, Iran; c Student Research Committee, School of Medicine, Shahroud University of Medical Sciences, Shahroud, Iran; d Student Research Committee, Shiraz University of Medical Sciences, Shiraz, Iran; e Sepehr Heart Center, Baharloo Hospital, Tehran University of Medical Science, Tehran, Iran; f School of Medicine, Ziaeian Hospital, International Campus, Tehran University of Medical Sciences, Tehran, Iran; g Department of Health Management, Policy and Economics, School of Public Health, Tehran University of Medical Sciences, Tehran, Iran

**Keywords:** Burden, Epidemiology, Ischemic heart disease, MENA

## Abstract

**Introduction:**

Despite the burden of ischemic heart disease (IHD), there remains a paucity of research on the incidence, mortality, and burden of this condition in the Middle East and North Africa (MENA) regions. This study aimed to evaluate the epidemiology and the risk factors associated with IHD in the MENA region.

**Methods:**

This study was performed based on the GBD study 2019 data. We retrieved the data related to the epidemiology and burden of IHD, including prevalence, incidence, years of life lost due to premature death (YLLs), years lived with disability (YLDs), and mortality at the global level and in MENA countries across years and sexes.

**Results:**

IHD accounted for approximately 2.55 million (95 % UI 2.29–2.83) incident cases in MENA in 2019, with an age-standardized incidence rate of 613.87 (95 % UI 555.84–675.16) per 100 000 people, which has decreased by 9 % between 1990 and 2019. IHD accounted for 11.01 % of DALYs causes in MENA in 2019, an increase of 68 % compared to 1990. The DALYs rate from IHD increased with age in both men and women and was higher in men than in women in all age groups, except 85–89 years age-group, in 2019.

**Conclusion:**

The age-standardized prevalence and incidence of IHD are decreasing in MENA. However, this reduction is lower than the global level, which can be due to a weaker performance of the countries in the region in reducing the prevalence and incidence of the disease compared to the global average.

## 1 Introduction

Cardiovascular diseases (CVD) account for roughly 33 % of global mortality [1]. The prevalence of CVD has significantly increased, from 271 million cases in 1990 to 523 million cases in 2019 [2]. The worldwide economic burden of CVD was estimated to be approximately 863 billion USD in 2010, projected to increase to 1044 billion USD by 2030 [3]. Ischemic heart disease (IHD) is one of the major CVDs. The principal pathological mechanism responsible for IHD is atherosclerosis, an inflammatory ailment of the arteries linked to lipid accumulation and metabolic alterations resulting from various risk factors. Thus, IHD manifests clinically as myocardial infarction and ischemic cardiomyopathy [4]. IHD has been acknowledged as a significant challenge to achieving sustainable development in the current era [5]. The prevalence of chronic disabilities and impaired quality of life among individuals with nonfatal IHD is rising [6]. CVDs are often associated with several prevalent risk factors, including but not limited to high blood pressure, elevated low-density lipoprotein (LDL) cholesterol, diabetes, smoking and exposure to secondhand smoke, obesity, an unhealthy diet, and physical inactivity [7]. It is worth noting that the global increase in aging is a potential risk factor for IHD [8], [9].

The trend of diseases has shifted from infectious diseases to non-communicable diseases, including IHD, due to urbanization, especially in low- and middle-income countries. Despite advancements in the treatment of infectious diseases, this lifestyle change has significantly impacted disease patterns [10]. Given that the nations in the Middle East and North Africa (MENA) region tend to fall under the category of low and middle-income countries [11], it is imperative to ascertain the scope of the burden of IHD in this particular area. Notably, the average total cost of IHD care in low- and middle-income countries, expressed as country-specific health costs per capita, was 10 % of total health care costs [12].

Despite the significant impact of IHD on both individuals and society, there remains a paucity of research on the incidence, mortality, and burden of this condition in the MENA region. The present investigation was formulated utilizing the Global Burden of Diseases (GBD) database, which furnished comprehensive information on the epidemiological metrics of IHD spanning the period from 1990 to 2019. The study aimed to evaluate the epidemiology and the risk factors associated with IHD in the MENA region.

## 2 Methods

### 2.1 Overview & data source

This study was performed based on the GBD study 2019 data that was conducted by the Institute of Health Metrics and Evaluation (IHME). The estimation of burden and epidemiology of IHD was done based on the vital registration and verbal autopsy data worldwide; in the cases of high-quality vital registration data in a country or region, verbal autopsy data were outlain. The details of the GBD study methods are reported elsewhere [13]. We retrieved the data related to the epidemiology and burden of IHD, with B.2.2 cause code via the GBD results tool on the IHME website [14], at the global level and MENA countries, including Afghanistan, Algeria, Bahrain, Egypt, Iran, Iraq, Jordan, Kuwait, Lebanon, Libya, Morocco, Oman, Palestine, Qatar, Saudi Arabia, Sudan, Syria, Tunisia, Turkey, United Arab Emirates, and Yemen.

### 2.2 Indices

Crude numbers of IHD prevalence, incidence, years of life lost due to premature death (YLLs), years lived with disability (YLDs), and mortality across countries, age groups, and sexes were retrieved. Also, age-standardized rates per 100 000 people were reported for all these indices. 95 % uncertainty intervals were reported for all these values, and the changes in values from 1990 to 2019 were evaluated. We also reported disability-adjusted life years (DALYs) calculated by adding YLLs to YLDs.

All-cause DALYs of the countries in 1990 and 2019 were used to calculate the proportion of DALYs caused by IHD in these years; it was calculated by dividing the DALYs attributable to IHD to total all causes DALYs.

The sociodemographic index (SDI) was developed in the GBD study to evaluate regions’ development status; it was calculated based on lag distributed income per capita, the mean education years for adults older than fifteen, and the fertility rate of people younger than twenty-five. SDI ranges between 0 and 1; as the index increases, it shows higher development status.

We evaluated the association between DALYs and SDI in each country from 1990 to 2019. LOESS regression was used to determine the expected association between SDI and DALYs.

Analyses were completed with Python version 3.8.12 via the following libraries: Numpy 1.21.2, Pandas 1.3.3, Matplotlib 3.4.3, Folium 0.12.0, and Statsmodels 0.13.0.

## 3 Results

### 3.1 Incidence & prevalence

**Fig. 1:**
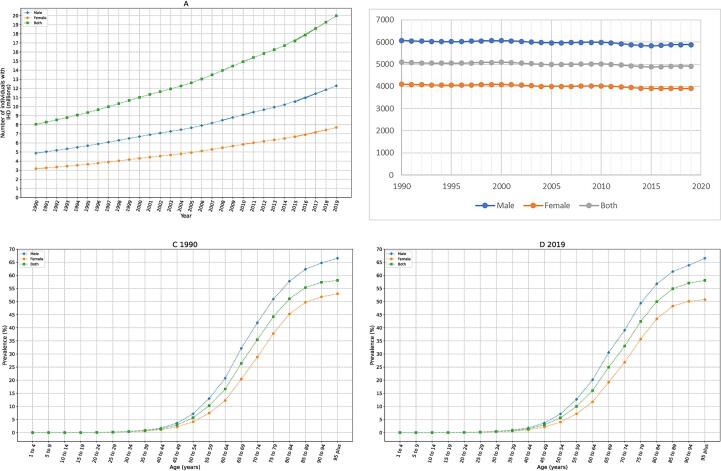
Prevalence of IHD in MENA. A. trend of overall and sex-specific numbers of IHD patients B. Age-standardized prevalence rate of IHD (per 100 000 people) C. Age- and sex-specific prevalence of IHD in 1990 D. Age- and sex-specific prevalence of IHD in 2019.

From 1990 to 2019, the number of living patients with IHD in MENA increased 2.48-fold, from 8.05 million to 19.98 million (Fig. 1A), while the corresponding age-standardized prevalence rate of IHD decreased 3.5 % from 5087.39 (95 % UI 4736.66–––5477.87) per 100 000 people to 4911.06 (4552.67– 5295.09) per 100 000 people. In addition, IHD accounted for approximately 1.08 million (95 % UI 9.73–12.01) incident cases in MENA in 1990, given the 2.55 million (95 % UI 2.29–2.83) incident cases in 2019, shows a 2.36-fold increase during these three decades. However, with an age-standardized incidence rate of 613.87 (95 % UI 555.84–675.16) per 100 000 people in 2019 in contrast to the age-standardized incidence rate of 674.52 (95 % UI 555.84–675.16) per 100 000 people in 1990, a decrease of 9 % is reported in age-standardized incidence rate of IHD in MENA between 1990 and 2019 (Table 1).

**Table 1:** Prevalent cases, incident cases and death for ischemic heart disease in 2019 for both sexes and percentage change of age-standardized rates by Global Burden of Disease 2019.

	**Prevalence (95 % uncertainty interval)**			**Incidence (95 % uncertainty interval)**			**Death (95 % uncertainty interval)**		
	**Counts** **(2019)**	**Age-standardized rate** **estimates** (**per 100 000 people)** **(2019)**	**Percentage change in** **age-standardized rates** **between** **1990 and 2019**	**Counts** **(2019)**	**Age-standardized rate** **estimates** (**per 100 000 people)** **(2019)**	**Percentage change in** **age-standardized rates** **between** **1990 and 2019**	**Counts** **(2019)**	**Age-standardized rate** **estimates** (**per 100 000 people)** **(2019)**	**Percentage change in** **age-standardized rates** **between** **1990 and 2019**
**Global**	197219449.808 [177688201.282–––219501074.494]	2421.023 [2180.504–––2692.646]	−4.6 [−5.7 - −3.6]	21203479.218 [18799321.606–––23704123.883]	262.394 [233.245–––293.257]	−17.1 [−17.9 - −16.2]	9137791.144 [8395682.479–––9743550.131]	117.952 [107.833–––125.918]	−30.8 [−34.8 - −27.2]
**MENA**	19979927.192 [18501725.438–––21563634.702]	4911.063 [4552.666–––5295.091]	−3.5 [−4.5 - −2.2]	2550431.562 [2287729.768–––2826389.560]	613.866 [555.840–––675.163]	−9 [−10.3 - −7.5]	799484.398 [706348.598–––909786.815]	219.011 [194.152–––246.754]	−29.2 [−36.9 - −21.8]
Algeria	1447170.069 [1337136.193–––1562324.552]	4581.404 [4248.861–––4938.675]	−5.4 [−8.2 - −2.4]	179174.190 [159670.926–––199668.652]	577.823 [525.249–––637.523]	−18 [–22.9 − −13.5]	58692.070 [47672.635–––71562.898]	237.255 [197.132–––282.690]	−40 [−51.7 - −26.5]
Bahrain	49533.891 [45551.810–––54048.115]	5212.930 [4824.345–––5645.002]	−4.6 [−7.5 - −1.8]	6461.239 [5474.787–––7565.760]	643.304 [556.877–––746.518]	−4.8 [−9 - −0.3]	863.355 [697.715–––1059.732]	155.095 [129.289–––186.669]	−64.9 [−71.4 - −55.9]
Egypt	3263745.927 [3033581.347–––3513427.752]	5623.945 [5255.286–––6014.876]	3.6 [0.6–––6.6]	450547.974 [411982.786–––491750.460]	759.927 [705.886–––819.385]	−2.8 [−7.9–––2.3]	181884.575 [138958.556–––233632.325]	359.267 [281.819–––447.027]	−12.5 [–32.4–––8.8]
Iran	4335510.092 [3950112.505–––4761788.189]	6198.454 [5644.356–––6814.639]	−0.8 [−1.7–––0.1]	593000.942 [515829.523–––676050.021]	829.138 [719.942–––945.224]	−7.7 [−9.5 - −6]	102798.782 [94454.767–––111215.263]	163.561 [148.984–––176.168]	−42.7 [−47.9 - −38.1]
Iraq	1141915.727 [1054978.539–––1233915.998]	5387.802 [4989.130–––5819.952]	−3.9 [−6.4 - −1]	140745.672 [125822.938–––156093.571]	644.427 [582.408–––711.605]	−8.6 [−13 - −4.3]	46847.942 [38262.597–––55510.862]	255.404 [214.105–––292.610]	−17.9 [–33.6 − −2]
Jordan	310387.925 [286476.800–––336917.292]	5112.563 [4730.610–––5526.306]	−2.3 [−4.8–––0.9]	37936.729 [32681.680–––43525.153]	603.644 [522.468–––687.549]	−5.9 [−10.2 - −1.8]	6110.958 [5195.842–––7285.931]	121.922 [103.146–––144.054]	−45.9 [−55 - −34.6]
Kuwait	136260.480 [125999.004–––147518.386]	5583.124 [5166.572–––6015.855]	3.4 [0.3–––6.8]	16837.076 [14644.446–––19358.708]	648.352 [559.179–––747.066]	2.2 [−2.4–––6.8]	2599.191 [2165.338–––3105.419]	108.535 [90.735–––129.196]	−44.2 [−52.7 - −34]
Lebanon	257386.167 [238945.227–––276978.440]	4949.351 [4595.368–––5330.647]	2.3 [−0.5–––5.3]	32025.023 [28354.949–––35958.074]	619.681 [547.831–––696.093]	−3.4 [−7.7–––0.7]	12251.420 [8865.728–––14092.289]	241.231 [174.107–––277.123]	−31.6 [−48.9 - −20.5]
Libya	234280.473 [217472.251–––253276.712]	4928.053 [4569.629–––5346.030]	8.5 [5.4–––11.7]	29205.148 [25486.670–––33143.187]	594.308 [515.855–––677.473]	7.6 [2–––13.4]	7826.805 [6177.052–––10362.771]	171.304 [135.447–––226.046]	−9.8 [−29.1–––14.7]
Morocco	1530982.533 [1412022.754–––1666262.388]	5223.563 [4834.831–––5652.989]	−1.9 [−4.8–––0.8]	190735.598 [173096.701–––211943.183]	644.782 [589.097–––707.248]	−4.4 [−9–––0.5]	72011.524 [56906.327–––84500.531]	278.540 [224.656–––321.408]	−9 [−25.2–––4.8]
Palestine	108744.102 [100339.187–––117399.769]	4962.816 [4601.543–––5349.499]	3.5 [0.5–––6.5]	13978.455 [12079.556–––16022.315]	623.640 [542.217–––706.331]	1.5 [−3–––6.1]	3809.825 [3325.695–––4366.034]	207.158 [180.071–––236.145]	−28.5 [−42.3 - −9.2]
Oman	92281.566 [85360.688–––100196.710]	5571.292 [5175.404–––5980.983]	12.2 [8.7–––16]	12346.346 [10823.747–––14054.609]	731.240 [639.405–––831.311]	11.4 [6.1–––17.4]	3411.646 [3045.722–––3828.428]	329.850 [296.028–––364.092]	−29.8 [−41.9 - −13]
Qatar	46196.419 [42120.704–––50305.419]	4851.453 [4485.765–––5220.832]	2.2 [−1.1–––5.3]	6439.719 [5455.860–––7538.192]	596.600 [512.228–––688.577]	0.6 [−4.2–––6.1]	830.008 [630.607–––1067.603]	252.986 [205.749–––305.427]	−37.7 [−50.2 - −20.8]
Saudi Arabia	835248.920 [771862.181–––903745.515]	5229.026 [4848.246–––5629.194]	12.4 [8.7–––16.4]	108673.267 [96040.908–––122753.072]	612.582 [545.387–––686.372]	7.7 [2–––13.8]	29689.065 [24089.216–––36175.781]	205.598 [172.875–––238.975]	−14.8 [–33.1–––9.6]
Syrian Arab Republic	586347.202 [544902.314–––633878.731]	5121.232 [4779.092–––5506.511]	5.1 [2.4–––8]	80332.227 [71888.247–––89817.809]	712.842 [644.734–––785.671]	4.5 [−0.8–––10.1]	33541.545 [26238.916–––43170.044]	359.717 [288.251–––449.747]	−7.2 [−28.4–––22.9]
Tunisia	546938.962 [506332.368–––589711.310]	4480.199 [4160.977–––4823.783]	2.3 [−0.7–––5.1]	68219.562 [60186.971–––77243.816]	558.099 [496.557–––627.102]	−0.5 [−5.6–––5]	21457.029 [16189.464–––27280.550]	193.454 [146.862–––244.040]	−19 [−38.5–––4.9]
Turkey	2785048.633 [2532629.953–––3082413.238]	3226.967 [2942.118–––3563.640]	–23.8 [−26.8 - −20.3]	281416.732 [252885.720–––312698.685]	325.484 [293.750–––359.905]	−31.4 [−34.9 - −27.4]	99046.377 [80454.391–––120867.092]	120.959 [97.994–––147.199]	−47.4 [−58.1 - −34.7]
United Arab Emirates	197600.390 [180763.318–––216932.557]	5246.718 [4863.166–––5666.822]	8.3 [4.9–––11.9]	27614.030 [23611.897–––32367.936]	654.003 [565.640–––754.135]	8 [3.3–––13.2]	4880.126 [3503.693–––6754.688]	175.396 [134.754–––223.386]	−41 [−54.1 - −24.8]
Yemen	592996.250 [547709.124–––641371.993]	4859.230 [4498.402–––5243.997]	2.8 [−0.4–––5.9]	79099.839 [70449.369–––88561.032]	636.712 [572.385–––709.068]	−3 [−7.8–––2.4]	32304.751 [26019.791–––42118.222]	294.763 [243.735–––374.516]	−16.7 [–32.3–––5.2]
Afghanistan	580637.745 [535688.020–––628879.746]	5203.628 [4814.574–––5625.489]	−2 [−5–––1.3]	79900.777 [71092.350–––89425.570]	672.223 [600.778–––751.084]	−7.7 [−12.6 - −2.8]	34627.787 [26988.785–––42664.911]	320.872 [253.973–––385.291]	−21.8 [−39 - −4.3]
Sudan	880414.430 [812353.709–––950642.826]	5091.095 [4705.996–––5471.101]	3.9 [0.9–––7.2]	113149.820 [101102.047–––126114.967]	636.363 [572.224–––705.453]	−7.3 [−11.8 - −2.3]	43187.356 [33612.674–––54936.109]	271.473 [214.925–––338.380]	−25 [−38.3 - −9]

The age-standardized prevalence rate was higher in men than in women between 1990 (6069.38 [5647.52– 6537.71] per 100 000 people in men and 4098.67 [3804.92 – 4411.37] per 100 000 people in women) and 2019 (5882.71 [5457.54 – 6353.14] per 100 000 people in men and 3908.29 [3632.52 – 4218.19] per 100 000 people in women; Fig. 1B). The age-specific prevalence of IHD increased with age is shown in Fig. 1C, D.

**Fig. 2:**
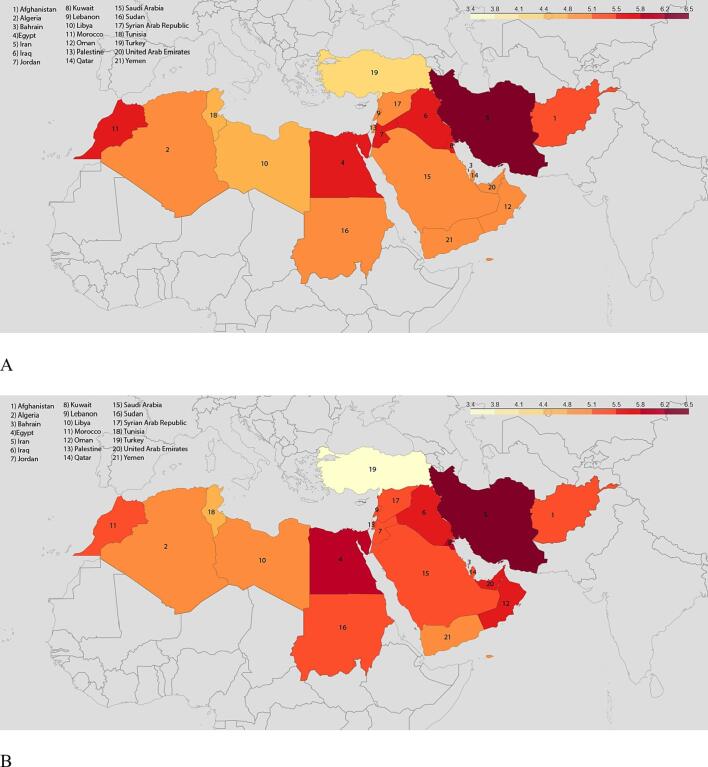

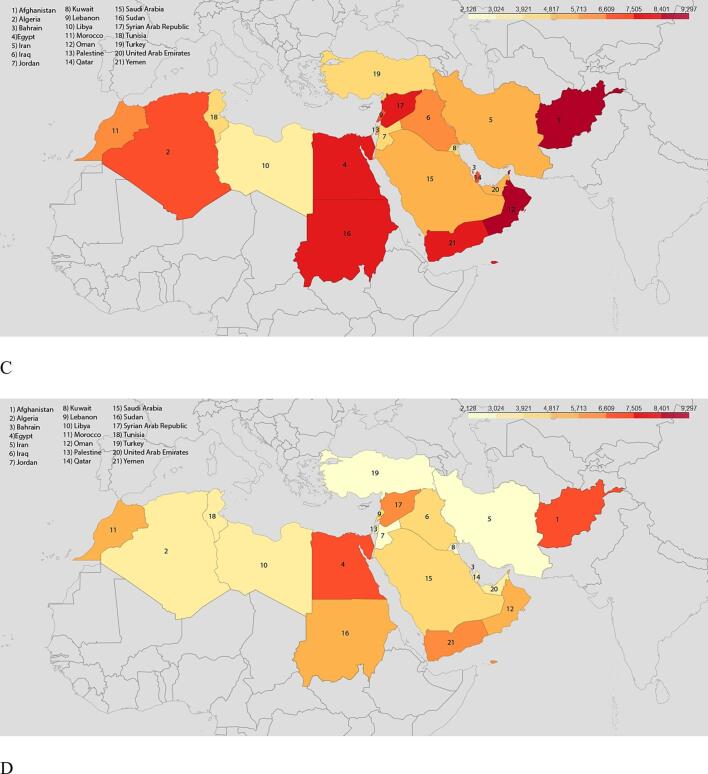
Geographical distribution of IHD in MENA. A. Age-standardized prevalence rates (thousand people per 100 000 people) in 1990B. in 2019. C. Age-standardized DALY rates per 100 000 people in 1990 D. in 2019.

Age-standardized prevalence rate varied notably throughout the MENA region (Fig. 2). Among the 21 countries, Iran had the highest age-standardized prevalence in both 1990 (6250.65 [5678.67 – 6852.20] per 100 000 people) and 2019 (6198.45 [5644.36 – 6814.64] per 100 000 people), whereas Turkey had the lowest in both 1990 (4237.28 [3902.21– 4604.87] per 100 000 people) and 2019 (3226.97 [2942.12 – 3563.64] per 100 000 people) (Fig. 2A, B).

From 1990 to 2019, Saudi Arabia showed the greatest relative increase (around 12.4 %) in the age-standardized prevalence rate of IHD, from 4650.49 [4301.58 to 5004.50] per 100 000 people to 5229.03 [4848.25 – 5629.19] per 100 000 people, whereas Turkey showed the largest relative decrease (around 23.8 %), from 4237.28 [3902.21 – 4604.87] per 100 000 people to 3226.97 [2942.12 – 3563.64] per 100 000 people (Appendix – Supplementary 1,2).

### 3.2 YLD & YLL

IHD contributed to 351.02 [234.24 – 494.90] thousands YLDs and 17.64 [15.26 – 20.45] million YLLs in MENA in 2019, an increase of 147 [141–153] percent, and 65 [42–87] percent in comparison with 1990, respectively; the corresponding age-standardized YLDs rate of IHD decreased 4 % from 87.36 [58.52 – 122.87] per 100 000 people in 1990 to 83.83 [56.46 – 117.15] per 100 000 people in 2019. In addition, IHD accounted for an age-standardized YLLs rate of 4075.12 [3561.25 – 4675.01] per 100 000 people in MENA in 2019, a decrease of 34 % compared with 1990 (Table 2).

**Table 2:** Years of life lost (YLL), Years of healthy life lost due to disability (YLD) and Disability-adjusted life years (DALYs) for ischemic heart disease in 2019 for both sexes and percentage change of age-standardized rates by Global Burden of Disease 2019.

	**YLLs (95 % uncertainty interval)**			**YLDs (95 % uncertainty interval)**			**DALYs (95 % uncertainty interval)**		
	**Counts** **(2019)**	**Age-standardized rate** **estimates** (**per 100 000 people)** **(2019)**	**Percentage change in** **age-standardized rates** **between** **1990 and 2019**	**Counts** **(2019)**	**Age-standardized rate** **estimates** (**per 100 000 people)** **(2019)**	**Percentage change in** **age-standardized rates** **between** **1990 and 2019**	**Counts** **(2019)**	**Age-standardized rate** **estimates** (**per 100 000 people)** **(2019)**	**Percentage change in** **age-standardized rates** **between** **1990 and 2019**
Global	176634915.822 [165028828.962–––188453376.898]	2177.014 [2033.997–––2321.466]	−29.1 [−34.1 - −24.6]	5395228.489 [3632732.670–––7581058.186]	66.531 [44.816–––93.539]	−6.2 [−8.3 - −4.1]	182030144.310 [170206778.294–––193504630.424]	2243.544 [2098.702–––2385.012]	−28.6 [–33.3 − −24.2]
MENA	17643805.379 [15257316.509–––20451536.599]	4075.116 [3561.255–––4675.008]	–33.7 [−42.1 - −25.3]	351016.528 [234240.226–––494897.998]	83.826 [56.458–––117.153]	−4 [−6.1 - −2.1]	17994821.907 [15580582.015–––20811862.229]	4158.941 [3650.736–––4751.708]	–33.3 [−41.4 - −25]
Algeria	1141474.636 [904537.763–––1438685.436]	3682.642 [2970.916–––4575.772]	−45.1 [−57.3 - −30.4]	26137.276 [17249.199–––36759.824]	80.469 [54.287–––113.298]	−1.2 [−6.3–––3.8]	1167611.912 [927773.512–––1464818.676]	3763.111 [3049.932–––4657.023]	−44.6 [−56.6 - −29.9]
Bahrain	22105.271 [17726.814–––27414.794]	2454.499 [2007.515–––2985.193]	−69.9 [−76 - −61.3]	1041.345 [690.077–––1478.366]	97.796 [66.390–––137.691]	−10.5 [−15.2 - −5.5]	23146.616 [18662.007–––28406.214]	2552.295 [2101.251–––3090.379]	−69.1 [−75.1 - −60.7]
Egypt	4364208.632 [3257227.140–––5733265.055]	6893.076 [5246.173–––8857.440]	−13.8 [−34.5–––9.8]	55729.576 [36568.184–––78859.637]	93.343 [62.376–––130.473]	5.3 [−0.1–––11.1]	4419938.208 [3313612.833–––5806683.587]	6986.419 [5361.902–––8973.940]	−13.6 [−34.1–––9.8]
Iran	1967791.139 [1837841.140–––2166854.649]	2761.748 [2570.407–––3020.773]	−48.4 [−53.6 - −44.2]	57633.239 [38661.730–––81196.411]	80.999 [55.047–––113.800]	−0.6 [−2.7–––1.2]	2025424.378 [1898317.784–––2228260.660]	2842.748 [2656.998–––3103.121]	−47.7 [−52.9 - −43.6]
Iraq	1049015.630 [825189.916–––1286971.937]	4675.562 [3771.296–––5614.034]	−24.3 [−41.1 - −5.6]	21916.320 [14776.852–––30526.425]	100.126 [67.535–––138.499]	−3.7 [−8.5–––1.4]	1070931.950 [845994.894–––1307890.395]	4775.688 [3863.589–––5709.059]	−24 [−40.6 - −5.5]
Jordan	141974.016 [118971.192–––170540.038]	2179.578 [1852.270–––2600.923]	−49.2 [−58.5 - −36.4]	5506.609 [3640.231–––7819.111]	85.645 [57.139–––119.623]	−3.8 [−8.4–––1.3]	147480.625 [124435.733–––177497.893]	2265.223 [1931.872–––2687.197]	−48.3 [−57.5 - −35.8]
Kuwait	69050.763 [56864.584–––83383.745]	2154.181 [1786.506–––2584.633]	−45 [−54.3 - –33.9]	2626.148 [1750.919–––3685.399]	98.011 [66.202–––136.856]	2.2 [−3.2–––7.8]	71676.910 [59488.763–––86115.334]	2252.193 [1881.575–––2687.812]	−43.9 [−52.9 - –33]
Lebanon	228923.309 [167535.282–––265747.984]	4406.459 [3224.739–––5112.732]	−35.5 [−51.4 - –22.2]	4448.296 [2971.527–––6123.797]	85.513 [56.982–––118.252]	6.5 [1.2–––12.2]	233371.606 [171955.126–––270077.001]	4491.972 [3316.327–––5196.531]	−35 [−50.4 - −21.9]
Libya	178677.662 [139408.763–––242636.931]	3338.569 [2611.073–––4461.047]	−11 [−31.1–––16.9]	4480.995 [3014.542–––6285.559]	90.401 [61.098–––125.805]	13.3 [7.2–––19.4]	183158.657 [144661.894–––247389.157]	3428.970 [2699.300–––4556.738]	−10.5 [−30.2–––16.8]
Morocco	1522233.032 [1164291.105–––1863727.554]	5036.534 [3915.779–––5983.341]	−16.3 [–33.6 − −0.2]	28789.535 [19241.415–––40789.416]	95.294 [64.736–––134.439]	−3.5 [−8.3–––1.6]	1551022.566 [1191648.773–––1885658.242]	5131.827 [4026.016–––6088.909]	−16.1 [–33.1 − −0.3]
Palestine	84690.572 [73163.936–––97776.320]	3702.041 [3228.790–––4246.441]	–33.6 [−47.8 - −14.1]	2013.948 [1343.489–––2848.144]	86.535 [58.120–––121.166]	2 [−3–––7.4]	86704.520 [75038.218–––99657.100]	3788.576 [3300.108–––4337.982]	–33 [−47.3 - −13.7]
Oman	81262.992 [70632.413–––93737.492]	5313.012 [4778.149–––5900.466]	−41.2 [−52.4 - −24.7]	1950.454 [1293.308–––2804.191]	104.441 [70.292–––146.102]	23.4 [15.3–––32.4]	83213.446 [72529.345–––95689.935]	5417.453 [4890.812–––6023.605]	−40.6 [−51.9 - −24.2]
Qatar	23088.212 [17175.951–––30233.500]	3495.721 [2778.317–––4304.402]	−50.5 [−61.7 - −36.1]	1132.183 [738.448–––1597.818]	101.468 [68.618–––140.674]	−2.1 [−10.4–––8]	24220.394 [18354.904–––31467.303]	3597.190 [2863.861–––4394.893]	−49.8 [−60.7 - −35.4]
Saudi Arabia	865769.726 [685500.154–––1084724.862]	4119.040 [3388.088–––4922.248]	−13.2 [-34.6–––14.4]	17789.735 [11862.064–––25220.135]	102.780 [69.100–––142.802]	18.5 [11.9–––25.9]	883559.461 [702672.545–––1099539.688]	4221.820 [3508.401–––5008.792]	−12.6 [–33.7–––14.5]
Syrian Arab Republic	753135.595 [573435.712–––991118.489]	6379.431 [4968.274–––8266.419]	−18.1 [-39.1–––12.4]	11883.456 [8058.064–––16688.906]	100.329 [68.996–––139.513]	8.1 [2.3–––14.4]	765019.051 [586984.154–––1001671.780]	6479.761 [5077.379–––8364.955]	−17.8 [-38.7–––12.2]
Tunisia	398833.141 [295002.975–––520067.596]	3277.837 [2445.197–––4246.520]	–22.8 [-43.3–––3.1]	9854.681 [6635.617–––13599.967]	79.541 [53.762–––110.103]	2.3 [-3–––8.2]	408687.822 [304295.765–––531699.785]	3357.378 [2517.601–––4339.073]	–22.4 [-42.6–––3]
Turkey	1788334.836 [1438804.510–––2191856.466]	2060.856 [1663.468–––2520.689]	−56.1 [-65.9 - −44.2]	58708.931 [39370.439–––83408.792]	67.526 [45.310–––95.912]	−26.9 [–32.7 - −21]	1847043.767 [1491706.838–––2245824.299]	2128.382 [1728.753–––2583.491]	−55.6 [-65 - −43.8]
United Arab Emirates	170485.686 [120425.182–––239663.918]	3282.994 [2468.749–––4305.434]	−39.7 [-54.9 - −20.5]	3906.651 [2559.284–––5699.689]	87.133 [58.876–––123.201]	10 [4–––16.6]	174392.337 [124283.748–––243499.793]	3370.127 [2554.965–––4399.532]	−39 [-54.1 - −19.9]
Yemen	796062.056 [610553.556–––1072202.485]	5780.234 [4599.899–––7576.157]	–22.4 [-39.4–––3.6]	10149.023 [6788.833–––14533.891]	79.547 [53.700–––110.562]	8.4 [2.7–––14.6]	806211.079 [620074.285–––1081163.144]	5859.781 [4694.646–––7645.436]	–22.1 [-38.9–––3.6]
Afghanistan	974916.205 [740862.127–––1268716.235]	6805.161 [5309.898–––8399.901]	−26.2 [-45 - −5.6]	9313.956 [6210.275–––13469.544]	78.341 [52.742–––109.476]	2.3 [-3.1–––7.7]	984230.160 [750693.251–––1275467.352]	6883.501 [5387.211–––8475.384]	−26 [-44.6 - −5.5]
Sudan	1003846.442 [755213.083–––1333048.104]	5202.836 [4015.990–––6679.169]	−31.1 [-45.7 - −11.9]	15647.545 [10458.674–––22395.553]	87.260 [58.608–––122.765]	14.6 [8.5–––21.5]	1019493.987 [771848.593–––1347945.110]	5290.096 [4103.351–––6787.133]	−30.6 [-45.1 - −11.6]

Age-standardized YLD rate varied throughout the MENA countries. Among the 21 countries, Bahrain had the highest age-standardized YLD rate in 1990 (109.31 [73.62 – 153.91] per 100 000 people), and Oman in 2019 (104.44 [70.29 – 146.10] per 100 000 people), whereas Yemen had the lowest in 1990 (73.36 [48.94 – 104.97] per 100 000 people) and Turkey in 2019 (67.53 [45.31 – 95.91] per 100 000 people). It was also revealed that Afghanistan had the highest age-standardized YLL rate in 1990 (9220.58 [7326.28 – 11535.66] per 100 000 people and Egypt in 2019 (6893.08 [5246.17 – 8857.44] per 100 000 people); while Libya had the lowest in 1990 (3752.04 [3062.05 – 4754.93] per 100 000 people) and in Turkey in 2019 (2060.86 [1663.47 – 2520.69] per 100 000 people).

From 1990 to 2019, Oman showed the greatest increase (23 %) in age-standardized YLD rate, whereas Turkey showed the largest decrease (27 %). The age-standardized YLL rate was decreased in all MENA countries during the study period ranging from 11 % in Libya to 70 % in Bahrain.

### 3.3 DALY

**Fig. 3:**
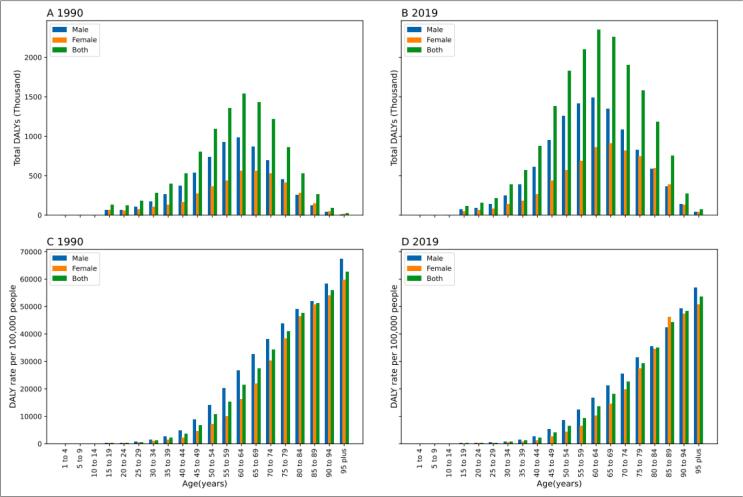
IHD burden by age. A. Age-specific DALYs by sex in 1990B. in 2019. C. Age-specific DALY rate per 100 000 by sex in 1990 D. in 2019.

IHD accounted for 11.01 % of DALYs causes in MENA in 2019, an increase of 68 % compared to 1990; while this proportion in global scale is 7.19 % in 2019 showing an increase of 54 % in comparison with 1990. Total DALYs due to IHD increased by more than 1.66-fold, from 10.86 million [95 % UI 10.04 – 11.73] in 1990 to 17.99 million [15.58 – 20.81] in 2019. Total DALYs count was higher in males (6.65 million [6.13 – 7.28] in 1990 and 11.03 million [9.51 – 12.91] in 2019) than in females (4.21 million [3.89 – 4.59] in 1990 and 6.96 million [5.99 – 8.03] in 2019; Appendix – Supplementary 3. The total number of DALYs due to IHD increased with age until 60–64 years old in both 1990 and 2019 (Fig. 3A–B), and then declined.

Age-standardized DALY rate from IHD decreased by 33 %, from 6232.35 [95 % UI 5783.40 – 6705.69] per 100 000 people in 1990 to 4158.94 [3690.74 – 4751.71] per 100 000 in 2019. Among the 21 MENA countries, Afghanistan had the highest age-standardized YLD rate (9297.15 [7407.11 – 11625.33] per 100 000) and Libya the lowest (3831.86 [3154.32 – 4834.38] per 100 000) in 1990, while in 2019, Egypt had the highest and Turkey the lowest with 6986.42 [5361.90 – 8973.94] per 100 000, and 2128.38 [1728.75 – 2583.49] per 100 000, respectively. (Fig. 2C-D; Appendix – Supplementary 4,5).

The DALYs rate from IHD increased with age in both men and women and was higher in men than in women in all age groups in both 1990 and 2019; the only exception is the 85–89-year age group in which females have a higher DALYs rate compared with males in 2019. (Fig. 3C, D).

The change in age-standardized DALYs rate from 1990 to 2019 was variable among countries; the highest relative decrease was in Bahrain (69 %) and the lowest in Libya (11 %) (See Appendix – Supplementary 4,5.).

In 1990, the age-standardized DALYs due to IHD accounted for 12.63 % of all-cause DALYs in MENA and showed substantial geographical variability (from 10.69 % in Sudan to 21.58 % in Bahrain). By 2019, the proportion of all-cause age-standardized DALYs caused by IHD had increased by 0.73 % to 13.36 % in MENA overall, ranging from 8.96 % in Turkey to 21.16 % in Syrian Arab Republic. (Table 3).

**Table 3:** Mean (95% uncertainty interval) proportion of all-cause age-standardized DALYs caused by ischemic heart disease, by country (1990 and 2019).

	1990	2019
Global	6.28	6.83
MENA	12.63	13.36
Algeria	15.26	13.94
Bahrain	21.58	10.45
Egypt	15.28	20.5
Iran	11.62	11.36
Iraq	13.45	15.13
Jordan	12.51	9.61
Kuwait	13.53	11.16
Lebanon	17.51	17.37
Libya	11.02	12.2
Morocco	13.1	16.59
Oman	20.75	18.78
Palestine	13.94	13.22
Qatar	19.25	14.03
Saudi Arabia	11.56	14.74
Syrian Arab Republic	19.29	21.16
Tunisia	12.44	13.92
Turkey	11.3	8.96
United Arab Emirates	13.96	11.66
Yemen	11.22	13.25
Afghanistan	10.76	12.42
Sudan	10.69	14.02

### 3.4 Death

IHD accounted for 25.77 % of death causes in MENA in 2019, an increase of 47 % compared to 1990; while this proportion in global scale is 16.17 % in 2019 showing an increase of 32 % in comparison with 1990. Total death number attributable to IHD in MENA in 2019 was 799.48 [706.35 – 909.79] thousands, an increase of 80 % from 1990; while age-standardized death rate was 219.01 [194.15 – 246.75] per 100 000 people in 2019 which showed 29 % decrease compared to 1990. The age-standardized death rate was higher in males in both 1990 and 2019. The male to female ratio of the age-standardized death rate was 1.30 and 1.24 in 1990 and 2019, respectively.

Oman had the highest age-standardized death rate attributed to IHD in 1990 (470.15 [382.97 – 562.72] per 100 000 people) and Libya had the lowest 189.88 [153.37 – 236.74] per 100 000 people); while in 2019 the highest and lowest rates were observed in Syrian Arab Republic (359.72 [288.25 – 449.75] per 100 000 people) and Kuwait (108.53[90.73 – 129.20] per 100 000 people), respectively (Fig. 5A, B).

The age-standardized death rate decreased in all MENA countries during the study period. The highest and lowest changes were seen in Bahrain (65 %) and Syrian Arab Republic (7 %), respectively.

### 3.5 SDI

**Fig. 4:**
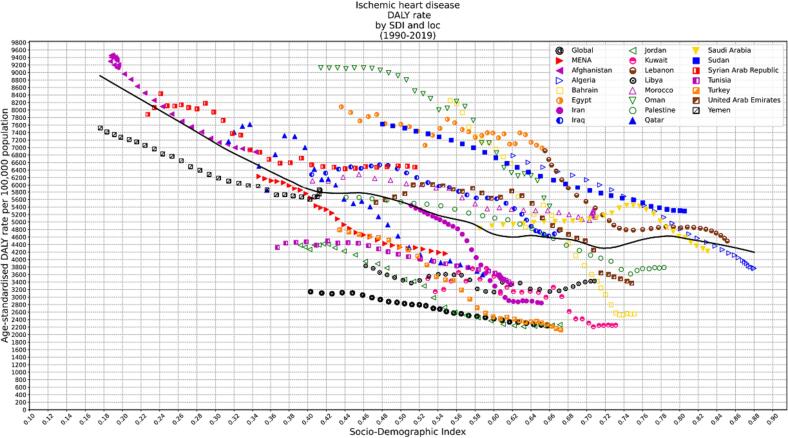
DALY rates for IHD in MENA by Sociodemographic Index (SDI), 1990–2019; Thirty points are plotted for each country and show observed age-standardized DALY rates per 100 000 people from 1990 to 2019 for that country.

Generally, there was a negative association between the age-standardized DALY rate and SDI between 1990 and 2019 (Fig. 4).

**Fig. 5:**
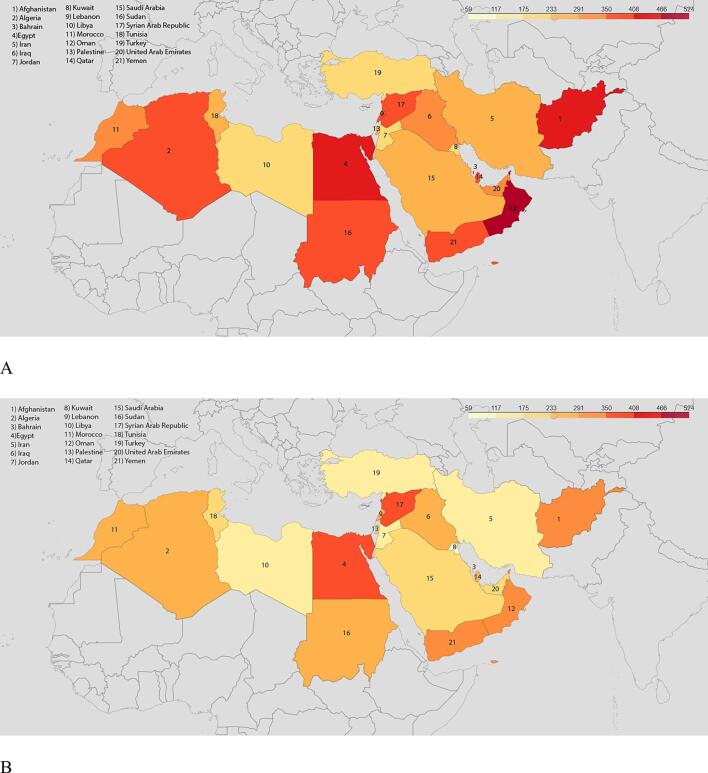
Geographical distribution of age-standardized death rate (per 100 000 people) attributable to IHD in MENA A. in 1990B. in 2019.

### 3.6 Attributable risk factors

The percentage of age-standardized DALY rates due to IHD attributable to the quantified risk factors in GBD 2019 differed in MENA countries. High Systolic blood Pressure, Dietary risks, and high LDL cholesterol were associated with the highest DALYs and Deaths among all. Table 4, Table 5 demonstrate percentages of age-standardized DALYs and death of IHD attributable to the most common IHD risk factors in MENA. Notably, the percentage contribution of alcohol to age-standardized IHD DALYs and deaths were −0.4 %, and – 0.3 %, respectively.

**Table 4:** Contributions of major risk factors in North Africa and Middle East region to age-standardized DALYs of ischemic heart disease, 2019.

A. Male


B. Female

**Table 5:** Contributions of major risk factors in North Africa and Middle East region to age-standardized Death of ischemic heart disease, 2019.

A. Male


B. Female

## 4 Discussion

The findings of this study showed that in overall the prevalence and incidence of IHD in the Middle East and North Africa have been increasing over the past 30 years, while the age-standardized prevalence and incidence rates have been decreasing. The 2.48-fold increase in the prevalence and 2.36-fold increase in the incidence of IHD indicate the need to provide more resources to control the disease. The main reason for this massive jump in the prevalence of IHD is undoubtedly an approximately 80 % growth in the MENA population from about 290 million in 1990 to around 530 million in 2019, with an increase of 10 years in life expectancy at birth, which results in a higher incidence of IHD [15]. In the past 50 years, the causes of death have significantly changed worldwide from nutritional deficiencies and infectious diseases to non-communicable diseases that lead to ischemic heart disease [16]. This epidemiological transformation began in high-income countries and has become a global epidemic over the past recent years. There has been a tremendous increase in the rate of ischemic heart disease in the Middle East and North Africa (MENA) region. Between 1990 and 2010, ischemic heart disease replaced lower respiratory tract infections as the leading cause of death in the Arab world, accounting for 14.3 % of deaths [17]. The growth rate of cardiac deaths is genuinely alarming. This rapid increase in coronary artery disease in a short period can be explained by lifestyle changes rather than genetic causes [18].

Although the findings of this study show that the age-standardized prevalence and incidence rate of IHD are decreasing, it is noteworthy that the rate of this decrease in the Middle East and North Africa region is lower than the rate at the global level. It confirms the weaker performance of the countries in the region in reducing the prevalence and incidence of the disease compared to the global average. There appears to be a significant association between Western dietary patterns and lifestyle with increased risk of IHD and associated risk factors among adults in the MENA region. On the contrary, increasing adherence to Mediterranean nutritional habits or their dietary components is associated with a reduced risk of IHD and related risk factors, which could be factors of this issue [19].

The prevalence of IHD is higher in men than in women. This finding is consistent with the previous study. In general, cardiovascular diseases, as the most important cause of death in the world, have noticeable sex differences [20]. Also, in the United States, the prevalence of IHD is higher in men than in women (7.4 % and 5.3 % in adults, respectively), and the mortality of IHD is up to 5 times higher in men [21]. In addition to the known hormonal effects in women, it should be emphasized that women follow a modified lifestyle and use more primary care than men [22]. Also, among the causes, in addition to the possibility of the female sex being a risk factor, it should be noted that women appear more often than men with signs and symptoms of IHD in the absence of obstructive coronary arteries, and diagnosis may be underestimated or not included in clinical studies [23], [24]. While men often experience the classic symptoms of chest pain radiating down the back and arms, women often experience “atypical” symptoms such as shortness of breath, chest pain at rest, nausea, and back pain. These symptoms are not easily recognized as diagnostic for IHD [25].

In MENA, the highest age-standardized prevalence rate is shown in Iran, and the highest relative increase in the last 30 years is related to Saudi Arabia. The high prevalence of dyslipidemia, diabetes and hypertension are the causes and risk factors, justifying the high prevalence of IHD in Iran [26], [27], [28]. Therefore, primary and secondary prevention of IHD, including lifestyle modification and dietary interventions in the Iranian population, is strongly recommended to reduce IHD and prevent its increase in Iran [29]. Regarding Saudi Arabia, the results of recent studies by Farid Mahmoud confirm that physical inactivity and unhealthy diet are common among Saudi adult citizens, which leads to the high prevalence of obesity, high blood pressure, and diabetes [30].

While YLL and YLD are decreasing in the Middle East and North Africa based on the age-standardized rate, the decrease in YLD is lower than the global rate. Also, some countries have an increasing trend in this regard, with the most significant increase in Oman. This is consistent with the findings of previous studies, which show that Oman has experienced the largest increase in the prevalence of obesity in the region. This increase is expected to continue until at least 2050 [31]. In addition, Oman has experienced a significant increase in life expectancy from 50 to 73.9 in nearly four decades following the demographic transition of the 1970 s [32]. Oman is one of only nine countries worldwide that has experienced an increase in life expectancy for men and women over 35 years since the 1950 s [33]. Also, the highest age-standardized rate of YLL in 2019 belongs to Egypt, which is why this country had the highest DALY rate in 2019. The quality of providing health services in cases related to cardiac issues in this country could be better, and so far, limited interventions have been made to improve it [34]. Special attention should be paid to disease management and prevention in this country due to the high rate of YLL and DALY.

It is also noteworthy that the highest relative decrease in the age-standardized rate of IHD prevalence during the 30 years of the study and the lowest age-standardized rate of prevalence, YLL, YLD, and DALY in 2019, are seen in Turkey. Previous studies show that Turkey has been able to reduce some of the critical risk factors of IHD, such as a more than 40 % reduction in smoking in men and the national tobacco control program, including the ban on smoking in public places, education, and awareness [35]. Also, diabetes and obesity control programs have been successful so far. The consumption of vegetables is traditionally present in the diet of this country. Following the economic development since 1990, it has been possible to consume more fruits and vegetables for most population classes [36], [37]. Healthy diets have a proven relationship with reducing systolic pressure, leading to reducing IHD. Also, studies have shown that modern medical treatment in this country has accounted for 47 % of the reduction in death caused by IHD [36].

DALY is higher in men in all age groups except 85 to 89, which can confirm the protective effect of the hormone that loses its impact after menopause [22]. Also, the contribution of age-standardized DALYs due to IHD to the total DALY causes in the Middle East is about twice the global average. In a country like the Syrian Arab Republic, this amount has reached more than one-fifth of the DALY causes of all diseases. A similar pattern is seen for IHD mortality, with age-standardized mortality rate in the Middle East and North Africa about twice the global average, and Syria having the highest rate in 2019. This issue shows the lack of proper attention to the disease and the years associated with high disability and mortality for IHD patients in the region, which calls for special attention to the prevention, treatment, and quality of treatment for these patients.

The risk factors for ischemic heart disease were investigated in this study. GBD risk variables at Level 2 were used. High systolic blood pressure, Dietary risks, High LDL cholesterol, High fasting plasma glucose, High body-mass index, Air pollution, Tobacco, Kidney dysfunction, Low physical activity, Non-optimal temperature, and Alcohol use, respectively, were the major risk factors for age-standardized death of IHD in MENA region in 2019. Hypertension is more prevalent in middle and low-income countries, and most of the countries in the region fall into this category, compared to high-income countries, and on the other hand, hypertension control is poorer in middle and low-income countries [38]. One of the serious situations of higher prevalence of hypertension as a risk factor for IHD in the region can be linked to the use of salty foods [39]. Bread and dairy products are major salt sources, with high consumption in the region [40]. Canada has the lowest blood pressure rate. This country has a comprehensive program that includes hypertension prevention, diagnosis, risk assessment, and treatment to control blood pressure. This valuable strategy will help the region’s countries reduce hypertension, which is the leading cause of mortality from IHD. Many of these risk factors are related to the cultural context of the region’s population. People’s Carbohydrate and fat consumption is relatively high, which is compatible with the high prevalence of obesity [41], [42]. Physical inactivity among Arab adults and adolescents is relatively high [43]. Also, senior family members stay sedentary at home when younger relatives are expected to perform errands. So, Education and health programs aimed at educating MENA populations about the benefits of a healthier lifestyle will be critical in lowering the future burden of IHD.

Our study is not without limitations. First, although ICD codes are widely accepted for death certification, and the 2019 GBD methodology has significantly improved data quality, misclassification of IHD deaths cannot be avoided entirely due to cardiovascular disease complexity and associated diseases. Second, the lack of follow-up data on nonfatal IHD patients identified in cross-sectional studies may be a major challenge in accurately quantifying the burden of IHD. Third, sociocultural and ethnic differences are not accounted for by GBD study models. These differences are often associated with health behaviors and risk factors that affect the global burden of IHD. But in general, the data quality is favorable due to the effort to increase the accuracy of IHME modeling. Upgrading the registry systems of the region will help to complete the information of such studies.

In **Conclusion**, despite the increase in the prevalence and incidence of IHD in MENA in the last three decades, the age-standardized prevalence and incidence rates of IHD are decreasing. But it is noteworthy that this reduction in the Middle East and North Africa region is lower than the global level. It confirms the weaker performance of the countries in the region in reducing the prevalence and incidence of the disease compared to the global average. The prevalence of IHD is higher in men than women, which can be related to hormonal factors or/and less detection of symptoms in women. Among the 21 countries in the Middle East, in five countries, including Iran and Saudi Arabia with the highest prevalence and increasing prevalence of IHD, respectively, and the countries of Oman, Egypt, and Syria with the highest increase in YLDs, the highest DALYs, and the highest proportions of DALYs attributed to the IHD, respectively, special attention should be paid. Successful experiences in countries such as Turkey can be compelling.

## CRediT authorship contribution statement

**Mohammad Ahmadi:** Conceptualization, Software, Writing – original draft, Supervision, Project administration, Writing – review & editing. **Shana Ahadi:** Conceptualization, Writing – original draft, Data curation. **Mohammad Amin Khadembashiri:** Software, Formal analysis, Writing – original draft. **Mohamad Mehdi Khadembashiri:** Software, Formal analysis, Writing – original draft. **Mehrdad Mahalleh:** Writing – review & editing, Data curation. **Hani AziziKia:** Writing – original draft, Data curation. **Hamid Reza Zare:** Writing – original draft, Data curation. **Amir Sobh Rakhshan Khah:** Validation, Writing – review & editing. **Hamidreza Hekmat:** Validation, Writing – review & editing. **Rajabali Daroudi:** Visualization, Writing – review & editing. **Ali Akbari Sari:** Visualization, Writing – review & editing.

## Declaration of competing interest

The authors declare that they have no known competing financial interests or personal relationships that could have appeared to influence the work reported in this paper.

## References

[1] Mozaffarian D., Benjamin E.J., Go A.S., Arnett D.K., Blaha M.J., Cushman M. (2016). Heart disease and stroke statistics-2016 update: a report from the american heart association. Circulation.

[2] Roth G.A., Mensah G.A., Johnson C.O., Addolorato G., Ammirati E., Baddour L.M. (2020). Global Burden of Cardiovascular Diseases and Risk Factors, 1990–2019: Update From the GBD 2019 Study. J. Am. Coll. Cardiol..

[3] Bloom DE C.E., Jané-Llopis E. (2011).

[4] Zmysłowski A., Szterk A. (2017). Current knowledge on the mechanism of atherosclerosis and pro-atherosclerotic properties of oxysterols. Lipids Health Dis..

[5] The changing patterns of cardiovascular diseases and their risk factors in the states of India: the Global Burden of Disease Study 1990-2016. The Lancet Global health. 2018;6(12):e1339-e51. 10.1016/S2214-109X(18)30407-8 PMC6227386 30219317

[6] Moran A.E., Forouzanfar M.H., Roth G.A., Mensah G.A., Ezzati M., Murray C.J. (2014). Temporal trends in ischemic heart disease mortality in 21 world regions, 1980 to 2010: the Global Burden of Disease 2010 study. Circulation.

[7] Vaduganathan M., Mensah George A., Turco Justine V., Fuster V., Roth G.A. (2022). The Global Burden of Cardiovascular Diseases and Risk. J. Am. Coll. Cardiol..

[8] Paneni F., Diaz Cañestro C., Libby P., Lüscher T.F., Camici G.G. (2017). The Aging Cardiovascular System: Understanding It at the Cellular and Clinical Levels. J. Am. Coll. Cardiol..

[9] L. Ferrucci, F. Giallauria, J.M. Guralnik, Epidemiology of aging, Radiologic clinics of North America. 2008;46(4):643-52, v. 10.1016/j.rcl.2008.07.005 PMC2692491 18922285

[10] Sampasa-Kanyinga H., Lewis R.F. (2015). Frequent use of social networking sites is associated with poor psychological functioning among children and adolescents. Cyberpsychol. Behav. Soc. Netw..

[11] Guetat I., Serranito F. (2007). Income convergence within the MENA countries: A panel unit root approach. Q. Rev. Econ. Finance.

[12] Gheorghe A., Griffiths U., Murphy A., Legido-Quigley H., Lamptey P., Perel P. (2018). The economic burden of cardiovascular disease and hypertension in low- and middle-income countries: a systematic review. BMC Public Health.

[13] Vos T., Lim S.S., Abbafati C., Abbas K.M., Abbasi M., Abbasifard M. (2020). Global burden of 369 diseases and injuries in 204 countries and territories, 1990–2019: a systematic analysis for the Global Burden of Disease Study 2019. Lancet.

[14] GHDx I. GBD results tool. Availabe online: http://ghdx healthdata org/gbd-results-tool (accessed on 1 Jan 2022). 2022.

[15] United Nations, Department of Economic and Social Affairs, Population Division (2022). World Population Prospects 2022, Online Edition. Retrieved from: https://population.un.org/wpp/Download/Standard/MostUsed/.

[16] Omran A.R. (1971). The epidemiologic transition. A theory of the epidemiology of population change. Milbank Mem. Fund Q..

[17] Mokdad A.H., Jaber S., Aziz M.I.A., AlBuhairan F., AlGhaithi A., AlHamad N.M. (2014). The state of health in the Arab world, 1990–2010: an analysis of the burden of diseases, injuries, and risk factors. Lancet (london, England)..

[18] Okrainec K., Banerjee D.K., Eisenberg M.J. (2004). Coronary artery disease in the developing world. Am. Heart J..

[19] Aljefree N., Ahmed F. (2015). Association between dietary pattern and risk of cardiovascular disease among adults in the Middle East and North Africa region: a systematic review. Food Nutr. Res..

[20] Regitz-Zagrosek V. (2012). Sex and gender differences in health. Science & Society Series on Sex and Science. EMBO Rep..

[21] Organization WH. Cardiovascular diseases 2017 [Available from: https://www.who.int/news-room/fact-sheets/detail/cardiovascular-diseases-(cvds).

[22] Barker-Collo S., Bennett D.A., Krishnamurthi R.V., Parmar P., Feigin V.L., Naghavi M. (2015). Sex Differences in Stroke Incidence, Prevalence, Mortality and Disability-Adjusted Life Years: Results from the Global Burden of Disease Study 2013. Neuroepidemiology.

[23] AlBadri A., Wei J., Mehta P.K., Shah R., Herscovici R., Gulati M. (2017). Sex differences in coronary heart disease risk factors: rename it ischaemic heart disease!. Heart.

[24] Regitz-Zagrosek V., Oertelt-Prigione S., Prescott E., Franconi F., Gerdts E., Foryst-Ludwig A. (2016). Gender in cardiovascular diseases: impact on clinical manifestations, management, and outcomes. Eur. Heart J..

[25] Smaardijk V.R., Lodder P., Kop W.J., van Gennep B., Maas A., Mommersteeg P.M.C. (2019). Sex- and Gender-Stratified Risks of Psychological Factors for Incident Ischemic Heart Disease: Systematic Review and Meta-Analysis. J. Am. Heart Assoc..

[26] Maddah M. (2007). Obesity and dyslipidemia among young general physicians in Iran. Int. J. Cardiol..

[27] Azimi-Nezhad M., Ghayour-Mobarhan M., Parizadeh M.R., Safarian M., Esmaeili H., Parizadeh S.M. (2008). Prevalence of type 2 diabetes mellitus in Iran and its relationship with gender, urbanisation, education, marital status and occupation. Singapore Med. J..

[28] Haghdoost A.A., Sadeghirad B., Rezazadehkermani M. (2008). Epidemiology and heterogeneity of hypertension in Iran: a systematic review. Arch. Iran. Med..

[29] Ebrahimi M., Kazemi-Bajestani S.M., Ghayour-Mobarhan M., Ferns G.A. (2011). Coronary artery disease and its risk factors status in iran: a review. Iran. Red Crescent Med. J..

[30] Mahmood F.M. (2018). Prevalence and prevention of lifestyle-related diseases in Saudi Arabia. Int. J. Health Sci..

[31] Kilpi F., Webber L., Musaigner A., Aitsi-Selmi A., Marsh T., Rtveladze K. (2014). Alarming predictions for obesity and non-communicable diseases in the Middle East. Public Health Nutr..

[32] R.R. Hajjar, T. Atli, Z. Al-Mandhari, M. Oudrhiri, L. Balducci, M. Silbermann, Prevalence of aging population in the Middle East and its implications on cancer incidence and care, Ann. Oncology : Official J. Eur. Soc. Med. Oncol. 2013;24 Suppl 7(Suppl 7):vii11-24. 10.1093/annonc/mdt268 PMC3767158 24001758

[33] Dicker D., Nguyen G., Abate D., Abate K.H., Abay S.M., Abbafati C. (2018). Global, regional, and national age-sex-specific mortality and life expectancy, 1950–2017: a systematic analysis for the Global Burden of Disease Study 2017. Lancet.

[34] Wong R., Hathi S., Linnander E.L., El Banna A., El Maraghi M., El Din R.Z. (2012). Building hospital management capacity to improve patient flow for cardiac catheterization at a cardiovascular hospital in Egypt. Jt. Comm. J. Qual. Patient Saf..

[35] Ankara. National Tobacco Control Programme and Action Plan of Turkey 2008–2012 2008 [Available from: http://www.tkd-online.org/PDFs/tobacco_plan_en.pdf,.

[36] Unal B., Sözmen K., Arık H., Gerçeklioğlu G., Altun D.U., Şimşek H. (2013). Explaining the decline in coronary heart disease mortality in Turkey between 1995 and 2008. BMC Public Health.

[37] Erdem Y., Arici M., Altun B., Turgan C., Sindel S., Erbay B. (2010). The relationship between hypertension and salt intake in Turkish population: SALTURK study. Blood Press..

[38] Mills K.T., Stefanescu A., He J. (2020). The global epidemiology of hypertension. Nat. Rev. Nephrol..

[39] Kelishadi R., Gheisari A., Zare N., Farajian S., Shariatinejad K. (2013). Salt intake and the association with blood pressure in young Iranian children: first report from the middle East and north Africa. Int. J. Prev. Med..

[40] Al Jawaldeh A., Rafii B., Nasreddine L. (2019). Salt intake reduction strategies in the eastern mediterranean region. Eastern Mediterranean Health Journal = La Revue De Sante De La Mediterranee Orientale = Al-Majallah Al-Sihhiyah Li-Sharq Al-Mutawassit..

[41] Al-Sarraj T., Saadi H., Volek J.S., Fernandez M.L. (2010). Metabolic syndrome prevalence, dietary intake, and cardiovascular risk profile among overweight and obese adults 18–50 years old from the United Arab Emirates. Metab. Syndr. Relat. Disord..

[42] Galal O.M. (2002). The nutrition transition in Egypt: obesity, undernutrition and the food consumption context. Public Health Nutr..

[43] Sharara E., Akik C., Ghattas H., Makhlouf O.C. (2018). Physical inactivity, gender and culture in Arab countries: a systematic assessment of the literature. BMC Public Health.

