# Supplementary material for: Burden of ischemic heart disease in the Middle East and North Africa (MENA) and attributable risk factors: An epidemiological analysis from 1990 to 2019
Source: Int J Cardiol Heart Vasc. 2023 Dec 3;50:101316. doi: 10.1016/j.ijcha.2023.101316 (PMC10899721; doi:10.1016/j.ijcha.2023.101316)
Supplement: Supplementary data 1 [file mmc1.docx]

Supplementary Table 1.

| Supplementary: Age-standardized prevalence rate of IHD by countries and sex (1990 and 2019) | | | | | | | | | | | | | | | | | | |
| --- | --- | --- | --- | --- | --- | --- | --- | --- | --- | --- | --- | --- | --- | --- | --- | --- | --- | --- |
|  | 1990 | | | | | | | | | 2019 | | | | | | | | |
|  | Male | | | Female | | | Both | | | Male | | | Female | | | Both | | |
|  | Mean | LUI | UUI | Mean | LUI | UUI | Mean | LUI | UUI | Mean | LUI | UUI | Mean | LUI | UUI | Mean | LUI | UUI |
| Global | 3226.17 | 2908.12 | 3573.10 | 1980.44 | 1770.39 | 2208.72 | 2538.60 | 2282.61 | 2819.39 | 3007.47 | 2717.43 | 3328.87 | 1911.53 | 1708.95 | 2140.34 | 2421.02 | 2180.50 | 2692.65 |
| MENA | 6069.38 | 5647.52 | 6537.71 | 4098.67 | 3804.92 | 4411.37 | 5087.39 | 4736.66 | 5477.87 | 5882.71 | 5457.54 | 6353.14 | 3908.29 | 3632.52 | 4218.19 | 4911.06 | 4552.67 | 5295.09 |
| Afghanistan | 6251.52 | 5796.74 | 6725.09 | 4276.67 | 3954.72 | 4606.62 | 5311.03 | 4933.52 | 5707.68 | 6244.17 | 5761.04 | 6775.21 | 4270.97 | 3934.82 | 4644.23 | 5203.63 | 4814.57 | 5625.49 |
| Algeria | 5747.72 | 5316.12 | 6183.65 | 3936.55 | 3650.56 | 4243.69 | 4840.92 | 4499.27 | 5195.97 | 5392.15 | 4983.43 | 5833.80 | 3737.65 | 3456.68 | 4034.13 | 4581.40 | 4248.86 | 4938.67 |
| Bahrain | 6418.13 | 5898.03 | 6956.01 | 4367.66 | 4044.67 | 4715.56 | 5462.79 | 5049.91 | 5889.77 | 6012.59 | 5548.35 | 6521.41 | 4041.90 | 3731.29 | 4386.29 | 5212.93 | 4824.35 | 5645.00 |
| Egypt | 6381.66 | 5949.39 | 6875.61 | 4483.23 | 4183.64 | 4815.33 | 5430.83 | 5080.90 | 5837.53 | 6441.42 | 5977.68 | 6924.10 | 4645.84 | 4344.47 | 4974.87 | 5623.95 | 5255.29 | 6014.88 |
| Iran | 7457.34 | 6801.25 | 8162.31 | 4978.11 | 4489.43 | 5479.11 | 6250.65 | 5678.67 | 6852.20 | 7597.05 | 6922.83 | 8329.06 | 4815.78 | 4354.02 | 5316.38 | 6198.45 | 5644.36 | 6814.64 |
| Iraq | 6693.46 | 6163.22 | 7244.87 | 4573.29 | 4241.98 | 4942.04 | 6381.66 | 5949.39 | 6875.61 | 6467.38 | 5964.07 | 6992.33 | 4368.44 | 4046.21 | 4736.64 | 5387.80 | 4989.13 | 5819.95 |
| Jordan | 6227.56 | 5735.09 | 6761.55 | 4219.40 | 3893.25 | 4558.88 | 5232.81 | 4841.00 | 5649.97 | 6066.43 | 5596.39 | 6598.82 | 4060.03 | 3741.51 | 4386.45 | 5112.56 | 4730.61 | 5526.31 |
| Kuwait | 6152.25 | 5679.80 | 6662.57 | 4243.53 | 3921.05 | 4591.12 | 5401.96 | 4997.76 | 5817.10 | 6411.42 | 5913.09 | 6953.62 | 4358.00 | 4035.32 | 4699.75 | 5583.12 | 5166.57 | 6015.86 |
| Lebanon | 5824.65 | 5393.17 | 6285.20 | 3886.39 | 3591.39 | 4183.18 | 4839.41 | 4495.40 | 5191.07 | 6084.69 | 5626.79 | 6565.33 | 4017.58 | 3717.82 | 4339.35 | 4949.35 | 4595.37 | 5330.65 |
| Libya | 5344.13 | 4950.34 | 5796.17 | 3650.52 | 3366.95 | 3934.32 | 4542.50 | 4206.34 | 4897.67 | 5868.70 | 5428.00 | 6352.25 | 3955.02 | 3653.19 | 4279.23 | 4928.05 | 4569.63 | 5346.03 |
| Morocco | 6421.63 | 5927.18 | 6977.72 | 4268.52 | 3934.14 | 4623.53 | 5326.36 | 4922.56 | 5749.27 | 6300.70 | 5831.02 | 6844.29 | 4171.37 | 3850.90 | 4519.30 | 6500.36 | 6018.90 | 7022.51 |
| Oman | 5868.37 | 5406.94 | 6396.71 | 4004.25 | 3701.01 | 4326.13 | 4965.57 | 4595.62 | 5361.00 | 6500.36 | 6018.90 | 7022.51 | 4413.24 | 4082.66 | 4756.64 | 5571.29 | 5175.40 | 5980.98 |
| Palestine | 5810.98 | 5363.15 | 6280.46 | 3957.61 | 3644.48 | 4286.44 | 4793.02 | 4441.11 | 5174.00 | 5990.98 | 5518.75 | 6492.22 | 4033.05 | 3733.93 | 4362.12 | 4962.82 | 4601.54 | 5349.50 |
| Qatar | 5448.64 | 5021.11 | 5900.41 | 3671.07 | 3380.25 | 3974.51 | 4748.06 | 4386.95 | 5119.45 | 5278.01 | 4873.55 | 5691.07 | 3520.34 | 3252.28 | 3797.98 | 4851.45 | 4485.76 | 5220.83 |
| Saudi Arabia | 5375.92 | 4955.81 | 5797.58 | 3671.45 | 3405.11 | 3963.85 | 4650.49 | 4301.58 | 5004.50 | 6020.26 | 5574.83 | 6489.71 | 4039.67 | 3735.03 | 4359.29 | 5229.03 | 4848.25 | 5629.19 |
| Sudan | 5821.52 | 5391.28 | 6283.92 | 3883.86 | 3573.91 | 4194.24 | 4900.82 | 4549.13 | 5277.78 | 5967.80 | 5506.27 | 6423.85 | 4040.35 | 3731.89 | 4360.28 | 5091.09 | 4706.00 | 5471.10 |
| Syrian Arab Republic | 5774.50 | 5352.47 | 6221.15 | 3873.57 | 3588.42 | 4167.58 | 4874.20 | 4532.59 | 5228.47 | 6083.05 | 5661.08 | 6544.87 | 4098.89 | 3817.71 | 4417.54 | 5121.23 | 4779.09 | 5506.51 |
| Tunisia | 5194.52 | 4786.92 | 5622.09 | 3523.37 | 3244.12 | 3814.78 | 4381.58 | 4054.57 | 4722.00 | 5393.24 | 4983.93 | 5838.22 | 3620.16 | 3345.82 | 3898.81 | 4480.20 | 4160.98 | 4823.78 |
| Turkey | 5103.33 | 4674.89 | 5579.91 | 3459.44 | 3174.42 | 3775.66 | 4237.28 | 3902.21 | 4604.87 | 3825.34 | 3429.82 | 4301.19 | 2699.31 | 2485.28 | 2939.78 | 3226.97 | 2942.12 | 3563.64 |
| United Arab Emirates | 5542.00 | 5115.35 | 6007.53 | 3746.05 | 3461.82 | 4034.23 | 4844.28 | 4491.66 | 5223.09 | 5783.47 | 5352.90 | 6256.99 | 3878.50 | 3580.94 | 4183.21 | 5246.72 | 4863.17 | 5666.82 |
| Yemen | 5768.63 | 5342.94 | 6226.04 | 3837.93 | 3547.74 | 4139.92 | 4728.65 | 4392.82 | 5084.05 | 5827.55 | 5395.01 | 6294.52 | 3923.19 | 3621.55 | 4231.30 | 4859.23 | 4498.40 | 5244.00 |
| Changes* |  | | | | | |  | | | | | |  | | | | | |
| LUI and UUI is the 2·5 to 97·5 centile values of the 95% uncertainty intervals. *: Changes is the total mean values at 2019 compared to 1990. | | | | | | | | | | | | | | | | | | |

Supplementary Table 2.

| Supplementary: Prevalence number of IHD by countries and sex (1990 and 2019) | | | | | | | | | | | | | | | | | | |
| --- | --- | --- | --- | --- | --- | --- | --- | --- | --- | --- | --- | --- | --- | --- | --- | --- | --- | --- |
|  | 1990 | | | | | | | | | 2019 | | | | | | | | |
|  | Male | | | Female | | | Both | | | Male | | | Female | | | Both | | |
|  | Mean | LUI | UUI | Mean | LUI | UUI | Mean | LUI | UUI | Mean | LUI | UUI | Mean | LUI | UUI | Mean | LUI | UUI |
| Global | 55618285.63 | 49974412.89 | 61910398.26 | 41276313.09 | 36825686.86 | 46108922.48 | 96894598.73 | 87038657.54 | 107953342.76 | 113650780.82 | 102282038.22 | 126035009.87 | 83568668.99 | 74700049.48 | 93579949.15 | 197219449.81 | 177688201.28 | 219501074.49 |
| MENA | 4880767.19 | 4524621.22 | 5266869.69 | 3168596.43 | 2926882.56 | 3420699.09 | 8049363.62 | 7457889.72 | 8705147.43 | 12284642.15 | 11368061.60 | 13247357.18 | 7695285.04 | 7141891.19 | 8313322.01 | 19979927.19 | 18501725.44 | 21563634.70 |
| Afghanistan | 221768.59 | 204684.67 | 239837.65 | 132580.80 | 122415.71 | 143297.46 | 354349.39 | 328050.88 | 382610.05 | 340294.17 | 313785.17 | 368658.93 | 240343.58 | 221781.43 | 260615.45 | 580637.75 | 535688.02 | 628879.75 |
| Algeria | 322059.28 | 296359.74 | 348595.45 | 217843.92 | 200034.30 | 236961.46 | 539903.20 | 498669.64 | 583145.01 | 879673.79 | 809797.07 | 951853.62 | 567496.28 | 523377.97 | 615079.28 | 1447170.07 | 1337136.19 | 1562324.55 |
| Bahrain | 5973.87 | 5480.81 | 6534.57 | 3034.27 | 2799.49 | 3288.20 | 9008.15 | 8281.30 | 9797.72 | 36138.23 | 33085.15 | 39755.80 | 13395.66 | 12297.86 | 14608.96 | 49533.89 | 45551.81 | 54048.12 |
| Egypt | 858231.12 | 795186.26 | 928690.95 | 587452.64 | 546712.77 | 633740.57 | 1445683.76 | 1350105.31 | 1563529.61 | 2097122.09 | 1938901.77 | 2263130.79 | 1166623.83 | 1081661.62 | 1258648.77 | 3263745.93 | 3033581.35 | 3513427.75 |
| Iran | 941343.99 | 844849.08 | 1043645.45 | 547769.88 | 493328.27 | 605680.67 | 1489113.87 | 1340078.90 | 1647733.38 | 2674326.69 | 2431911.48 | 2929212.53 | 1661183.40 | 1507053.65 | 1835044.09 | 4335510.09 | 3950112.51 | 4761788.19 |
| Iraq | 239229.28 | 221131.30 | 259162.70 | 170390.64 | 157842.63 | 184550.57 | 409619.92 | 379740.04 | 442519.55 | 676028.34 | 623409.61 | 733933.32 | 465887.39 | 429532.72 | 504946.87 | 1141915.73 | 1054978.54 | 1233916.00 |
| Jordan | 38750.61 | 35519.23 | 42377.35 | 24494.20 | 22643.71 | 26428.92 | 63244.81 | 58383.85 | 68585.51 | 196294.68 | 180282.30 | 214002.30 | 114093.24 | 105020.07 | 123576.80 | 310387.92 | 286476.80 | 336917.29 |
| Kuwait | 21532.53 | 19847.70 | 23466.33 | 9008.76 | 8348.52 | 9699.54 | 30541.29 | 28232.74 | 33033.92 | 93669.08 | 86306.44 | 101758.62 | 42591.40 | 39422.99 | 45978.96 | 136260.48 | 125999.00 | 147518.39 |
| Lebanon | 61376.30 | 56553.62 | 66602.71 | 41151.57 | 38058.71 | 44410.78 | 102527.87 | 94825.56 | 110382.92 | 142330.68 | 131566.66 | 153681.59 | 115055.49 | 106637.26 | 124316.95 | 257386.17 | 238945.23 | 276978.44 |
| Libya | 49165.84 | 45424.17 | 53311.58 | 29666.36 | 27398.39 | 32017.71 | 78832.20 | 73044.78 | 84988.28 | 143181.38 | 132467.20 | 155240.14 | 91099.09 | 84352.61 | 98312.76 | 234280.47 | 217472.25 | 253276.71 |
| Morocco | 407876.19 | 374494.02 | 444237.52 | 270133.36 | 248249.24 | 293284.27 | 678009.56 | 626291.32 | 733177.73 | 921972.58 | 848411.58 | 1005865.49 | 609009.95 | 559984.58 | 662759.12 | 1530982.53 | 1412022.75 | 1666262.39 |
| Oman | 19086.09 | 17472.65 | 20860.53 | 10488.72 | 9699.47 | 11351.74 | 29574.81 | 27243.47 | 32103.01 | 64258.73 | 59294.31 | 70090.90 | 28022.84 | 25822.93 | 30293.95 | 92281.57 | 85360.69 | 100196.71 |
| Palestine | 21708.28 | 20011.69 | 23504.35 | 17606.73 | 16240.80 | 19049.64 | 39315.00 | 36426.00 | 42383.47 | 63533.14 | 58280.22 | 69020.39 | 45210.97 | 41843.03 | 48859.89 | 108744.10 | 100339.19 | 117399.77 |
| Qatar | 4053.27 | 3698.05 | 4453.82 | 1196.14 | 1105.51 | 1301.25 | 5249.41 | 4810.11 | 5737.49 | 38998.81 | 35453.52 | 42641.92 | 7197.61 | 6622.65 | 7823.25 | 46196.42 | 42120.70 | 50305.42 |
| Saudi Arabia | 167375.91 | 154147.50 | 181384.56 | 80910.26 | 75008.30 | 87290.55 | 248286.17 | 229825.23 | 267864.07 | 591899.71 | 544744.47 | 642750.12 | 243349.21 | 224378.89 | 264259.21 | 835248.92 | 771862.18 | 903745.52 |
| Sudan | 267672.68 | 247063.61 | 290365.25 | 157920.67 | 145166.94 | 170416.06 | 425593.35 | 393775.16 | 458519.56 | 565703.01 | 520364.73 | 610367.75 | 314711.42 | 290936.96 | 339819.15 | 880414.43 | 812353.71 | 950642.83 |
| Syrian Arab Republic | 147676.66 | 136355.94 | 159881.08 | 86808.80 | 80153.89 | 93525.99 | 234485.47 | 217437.46 | 252415.88 | 365079.08 | 338295.56 | 395338.26 | 221268.12 | 205144.28 | 240825.44 | 586347.20 | 544902.31 | 633878.73 |
| Tunisia | 128367.93 | 117797.95 | 139392.94 | 78399.26 | 71875.16 | 85148.45 | 206767.19 | 190700.07 | 223554.41 | 320242.97 | 294772.39 | 347500.29 | 226696.00 | 209243.62 | 244527.83 | 546938.96 | 506332.37 | 589711.31 |
| Turkey | 817626.46 | 744684.65 | 893994.81 | 607134.83 | 556433.12 | 661545.71 | 1540139.64 | 1379567.84 | 1733164.00 | 1244908.99 | 1145415.09 | 1354430.14 | 2785048.63 | 2532629.95 | 3082413.24 | 1540139.64 | 1379567.84 | 1733164.00 |
| United Arab Emirates | 15093.94 | 13756.77 | 16639.97 | 4515.91 | 4171.05 | 4890.91 | 19609.86 | 17973.51 | 21501.92 | 166098.68 | 151583.51 | 182706.73 | 31501.71 | 28936.88 | 34520.29 | 197600.39 | 180763.32 | 216932.56 |
| Yemen | 121515.64 | 111504.64 | 131624.01 | 87957.55 | 81290.03 | 94864.67 | 209473.19 | 193760.49 | 225562.99 | 355175.68 | 327372.56 | 383769.61 | 237820.57 | 219917.31 | 256825.47 | 592996.25 | 547709.12 | 641371.99 |
| Changes* |  | | | | | |  | | | | | |  | | | | | |
| LUI and UUI is the 2·5 to 97·5 centile values of the 95% uncertainty intervals. *: Changes is the total mean values at 2019 compared to 1990. | | | | | | | | | | | | | | | | | | |

Supplementary Table 3.

| Supplementary: Age-standardized incidence rate of IHD by countries and sex (1990 and 2019) | | | | | | | | | | | | | | | | | | |
| --- | --- | --- | --- | --- | --- | --- | --- | --- | --- | --- | --- | --- | --- | --- | --- | --- | --- | --- |
|  | 1990 | | | | | | | | | 2019 | | | | | | | | |
|  | Male | | | Female | | | Both | | | Male | | | Female | | | Both | | |
|  | Mean | LUI | UUI | Mean | LUI | UUI | Mean | LUI | UUI | Mean | LUI | UUI | Mean | LUI | UUI | Mean | LUI | UUI |
| Global | 405.30 | 361.24 | 452.06 | 239.88 | 213.10 | 267.49 | 316.40 | 282.16 | 352.31 | 333.49 | 297.00 | 371.88 | 198.45 | 176.41 | 221.21 | 262.39 | 233.25 | 293.26 |
| MENA | 791.25 | 718.67 | 870.01 | 554.97 | 502.05 | 612.81 | 674.52 | 612.20 | 740.46 | 708.06 | 639.57 | 780.67 | 514.45 | 465.31 | 566.98 | 613.87 | 555.84 | 675.16 |
| Afghanistan | 835.74 | 748.48 | 927.73 | 614.46 | 548.13 | 689.41 | 728.15 | 654.64 | 806.76 | 764.47 | 680.96 | 854.88 | 588.44 | 525.73 | 658.64 | 672.22 | 600.78 | 751.08 |
| Algeria | 806.49 | 728.39 | 889.73 | 607.00 | 547.55 | 676.73 | 704.92 | 639.53 | 774.87 | 632.72 | 574.10 | 706.61 | 526.07 | 477.40 | 583.22 | 577.82 | 525.25 | 637.52 |
| Bahrain | 770.30 | 658.36 | 885.20 | 558.22 | 482.93 | 643.62 | 675.42 | 583.42 | 768.99 | 718.37 | 616.08 | 833.17 | 517.03 | 443.34 | 599.25 | 643.30 | 556.88 | 746.52 |
| Egypt | 882.70 | 810.29 | 963.09 | 679.32 | 624.48 | 742.79 | 782.15 | 723.68 | 848.99 | 808.66 | 740.70 | 877.21 | 732.16 | 677.05 | 791.42 | 759.93 | 705.89 | 819.39 |
| Iran | 1077.52 | 935.55 | 1233.27 | 706.31 | 614.45 | 807.63 | 898.31 | 783.95 | 1026.45 | 1007.77 | 876.06 | 1147.12 | 652.03 | 568.70 | 743.19 | 829.14 | 719.94 | 945.22 |
| Iraq | 826.71 | 740.77 | 920.89 | 588.48 | 525.36 | 659.65 | 705.34 | 633.10 | 787.44 | 752.19 | 676.28 | 830.31 | 538.54 | 482.62 | 600.63 | 644.43 | 582.41 | 711.60 |
| Jordan | 736.14 | 634.88 | 844.74 | 540.53 | 465.24 | 616.22 | 641.66 | 555.09 | 731.87 | 690.93 | 594.46 | 795.04 | 504.63 | 439.62 | 580.69 | 603.64 | 522.47 | 687.55 |
| Kuwait | 704.60 | 604.68 | 815.08 | 513.88 | 441.88 | 595.16 | 634.60 | 546.48 | 731.26 | 729.76 | 627.81 | 843.63 | 532.41 | 453.70 | 616.05 | 648.35 | 559.18 | 747.07 |
| Lebanon | 763.82 | 672.76 | 860.31 | 521.08 | 459.59 | 596.36 | 641.67 | 566.01 | 724.82 | 757.05 | 669.78 | 849.97 | 506.91 | 444.04 | 572.16 | 619.68 | 547.83 | 696.09 |
| Libya | 631.75 | 539.89 | 731.30 | 460.62 | 393.86 | 530.13 | 552.58 | 475.13 | 635.68 | 686.97 | 593.33 | 791.50 | 498.68 | 429.37 | 572.69 | 594.31 | 515.85 | 677.47 |
| Morocco | 793.58 | 713.87 | 882.22 | 561.18 | 498.53 | 628.72 | 674.27 | 609.30 | 748.83 | 746.22 | 676.51 | 820.72 | 543.62 | 491.76 | 603.79 | 644.78 | 589.10 | 707.25 |
| Oman | 750.10 | 651.04 | 857.97 | 541.17 | 471.12 | 620.96 | 656.17 | 569.13 | 748.19 | 833.74 | 727.42 | 951.96 | 592.89 | 516.18 | 677.45 | 731.24 | 639.41 | 831.31 |
| Palestine | 732.07 | 634.70 | 842.54 | 519.44 | 448.55 | 596.86 | 614.47 | 530.79 | 702.60 | 729.16 | 626.77 | 836.99 | 522.82 | 457.08 | 596.91 | 623.64 | 542.22 | 706.33 |
| Qatar | 651.77 | 559.07 | 753.68 | 474.67 | 405.74 | 544.12 | 592.89 | 511.01 | 684.54 | 635.82 | 543.00 | 735.64 | 464.43 | 399.43 | 536.87 | 596.60 | 512.23 | 688.58 |
| Saudi Arabia | 638.44 | 558.48 | 725.44 | 467.39 | 406.83 | 531.69 | 568.57 | 499.12 | 643.50 | 687.65 | 611.54 | 772.74 | 499.04 | 439.32 | 564.90 | 612.58 | 545.39 | 686.37 |
| Sudan | 803.70 | 721.62 | 889.80 | 558.68 | 500.73 | 627.91 | 686.65 | 617.95 | 761.94 | 720.94 | 647.60 | 803.54 | 538.40 | 480.09 | 598.98 | 636.36 | 572.22 | 705.45 |
| Syrian Arab Republic | 785.66 | 696.83 | 879.00 | 568.01 | 504.80 | 638.55 | 681.87 | 610.39 | 758.02 | 806.15 | 720.88 | 892.82 | 623.31 | 563.86 | 691.43 | 712.84 | 644.73 | 785.67 |
| Tunisia | 655.63 | 577.71 | 741.73 | 463.22 | 404.43 | 526.74 | 561.05 | 493.94 | 632.63 | 658.38 | 584.58 | 744.67 | 463.28 | 408.35 | 522.15 | 558.10 | 496.56 | 627.10 |
| Turkey | 563.35 | 510.83 | 618.02 | 390.48 | 352.10 | 431.98 | 474.24 | 432.28 | 517.87 | 360.58 | 325.71 | 402.23 | 291.78 | 260.40 | 324.30 | 325.48 | 293.75 | 359.90 |
| United Arab Emirates | 669.36 | 571.05 | 781.36 | 490.35 | 418.45 | 567.10 | 605.71 | 522.32 | 703.14 | 700.75 | 604.60 | 803.42 | 517.57 | 445.41 | 597.64 | 654.00 | 565.64 | 754.13 |
| Yemen | 779.32 | 689.86 | 874.25 | 539.57 | 476.08 | 613.49 | 656.28 | 578.94 | 736.86 | 738.06 | 661.07 | 822.49 | 538.88 | 480.03 | 606.13 | 636.71 | 572.39 | 709.07 |
| Changes* |  | | | | | |  | | | | | |  | | | | | |
| LUI and UUI is the 2·5 to 97·5 centile values of the 95% uncertainty intervals. *: Changes is the total mean values at 2019 compared to 1990. | | | | | | | | | | | | | | | | | | |

Supplementary Table 4.

| Supplementary: Incidence number of IHD by countries and sex (1990 and 2019) | | | | | | | | | | | | | | | | | | |
| --- | --- | --- | --- | --- | --- | --- | --- | --- | --- | --- | --- | --- | --- | --- | --- | --- | --- | --- |
|  | 1990 | | | | | | | | | 2019 | | | | | | | | |
|  | Male | | | Female | | | Both | | | Male | | | Female | | | Both | | |
|  | Mean | LUI | UUI | Mean | LUI | UUI | Mean | LUI | UUI | Mean | LUI | UUI | Mean | LUI | UUI | Mean | LUI | UUI |
| Global | 6899923.17 | 6097129.26 | 7732711.38 | 4852104.66 | 4283557.18 | 5425187.24 | 11752027.84 | 10376503.59 | 13161208.36 | 12532034.43 | 11092085.60 | 14022524.81 | 8671444.79 | 7705187.22 | 9677404.27 | 21203479.22 | 18799321.61 | 23704123.88 |
| MENA | 654223.50 | 587043.85 | 723620.79 | 430453.51 | 384868.64 | 477644.46 | 1084677.01 | 972519.65 | 1201363.41 | 1534813.81 | 1375052.62 | 1709606.02 | 1015617.75 | 911177.96 | 1122474.35 | 2550431.56 | 2287729.77 | 2826389.56 |
| Afghanistan | 29965.67 | 26740.00 | 33501.56 | 19314.78 | 17124.09 | 21853.91 | 49280.45 | 44299.74 | 55119.09 | 45647.22 | 40393.93 | 51075.44 | 34253.55 | 30524.39 | 38508.17 | 79900.78 | 71092.35 | 89425.57 |
| Algeria | 43867.24 | 39027.36 | 48976.77 | 31286.73 | 27472.23 | 35332.70 | 75153.97 | 67093.71 | 83680.87 | 103532.77 | 92084.78 | 116781.19 | 75641.42 | 67388.56 | 84413.71 | 179174.19 | 159670.93 | 199668.65 |
| Bahrain | 761.06 | 654.80 | 877.66 | 390.64 | 337.70 | 446.51 | 1151.70 | 993.47 | 1316.32 | 4732.91 | 3979.14 | 5588.98 | 1728.32 | 1489.84 | 2001.02 | 6461.24 | 5474.79 | 7565.76 |
| Egypt | 121060.99 | 110011.85 | 133528.64 | 87467.22 | 79490.86 | 96301.35 | 208528.21 | 190882.26 | 228245.99 | 274364.81 | 248792.14 | 300903.09 | 176183.16 | 159347.40 | 193838.77 | 450547.97 | 411982.79 | 491750.46 |
| Iran | 140104.27 | 119779.29 | 162183.69 | 78076.41 | 68042.81 | 89474.31 | 218180.68 | 187230.98 | 250908.61 | 366349.22 | 317515.28 | 418667.13 | 226651.72 | 197594.53 | 258246.99 | 593000.94 | 515829.52 | 676050.02 |
| Iraq | 30220.79 | 26880.29 | 33807.91 | 22079.44 | 19656.62 | 24808.94 | 52300.23 | 46753.02 | 58385.29 | 82659.28 | 73428.86 | 92346.79 | 58086.39 | 51651.09 | 64886.06 | 140745.67 | 125822.94 | 156093.57 |
| Jordan | 4848.60 | 4157.07 | 5604.72 | 3161.92 | 2722.58 | 3627.50 | 8010.52 | 6925.92 | 9193.87 | 23642.51 | 20356.22 | 27174.11 | 14294.22 | 12400.36 | 16387.05 | 37936.73 | 32681.68 | 43525.15 |
| Kuwait | 2688.94 | 2320.84 | 3080.18 | 1126.51 | 987.70 | 1285.80 | 3815.45 | 3325.50 | 4349.37 | 11489.24 | 9910.48 | 13343.88 | 5347.84 | 4663.96 | 6076.10 | 16837.08 | 14644.45 | 19358.71 |
| Lebanon | 8181.41 | 7114.21 | 9260.05 | 5489.87 | 4814.03 | 6260.51 | 13671.28 | 11935.11 | 15540.84 | 17577.41 | 15567.38 | 19738.33 | 14447.61 | 12649.90 | 16317.23 | 32025.02 | 28354.95 | 35958.07 |
| Libya | 6088.15 | 5227.90 | 7017.50 | 3801.89 | 3278.01 | 4325.83 | 9890.04 | 8561.53 | 11332.38 | 17511.53 | 15130.42 | 20119.27 | 11693.61 | 10161.68 | 13359.11 | 29205.15 | 25486.67 | 33143.19 |
| Morocco | 50754.33 | 45305.16 | 56874.23 | 35775.14 | 31586.55 | 40314.77 | 86529.46 | 77550.02 | 96572.49 | 111405.54 | 99782.83 | 124636.35 | 79330.06 | 71027.13 | 88342.24 | 190735.60 | 173096.70 | 211943.18 |
| Oman | 2607.79 | 2256.13 | 3001.30 | 1426.84 | 1234.24 | 1631.14 | 4034.63 | 3488.63 | 4592.13 | 8626.25 | 7508.10 | 9858.04 | 3720.10 | 3245.01 | 4236.42 | 12346.35 | 10823.75 | 14054.61 |
| Palestine | 2758.55 | 2381.95 | 3165.62 | 2313.71 | 2001.43 | 2651.81 | 5072.25 | 4404.63 | 5788.32 | 8084.52 | 6880.40 | 9455.40 | 5893.94 | 5163.18 | 6692.42 | 13978.46 | 12079.56 | 16022.31 |
| Qatar | 562.02 | 482.24 | 655.40 | 160.82 | 141.06 | 183.10 | 722.84 | 623.18 | 835.39 | 5451.72 | 4609.92 | 6398.18 | 988.00 | 845.41 | 1140.49 | 6439.72 | 5455.86 | 7538.19 |
| Saudi Arabia | 21219.61 | 18617.02 | 23977.46 | 10549.46 | 9259.43 | 11950.16 | 31769.07 | 27971.33 | 35748.07 | 76705.75 | 67381.37 | 87162.44 | 31967.51 | 27876.49 | 36278.66 | 108673.27 | 96040.91 | 122753.07 |
| Sudan | 37102.67 | 33063.59 | 41421.49 | 22763.43 | 20300.15 | 25504.10 | 59866.10 | 53662.08 | 66644.62 | 70480.10 | 62705.49 | 79380.86 | 42669.72 | 38048.35 | 47421.02 | 113149.82 | 101102.05 | 126114.97 |
| Syrian Arab Republic | 20665.28 | 18302.98 | 23306.56 | 12605.03 | 11163.55 | 14135.90 | 33270.31 | 29441.75 | 37257.29 | 49033.79 | 43567.00 | 55069.35 | 31298.44 | 27830.39 | 35259.41 | 80332.23 | 71888.25 | 89817.81 |
| Tunisia | 16212.16 | 14131.50 | 18458.30 | 10282.29 | 8969.81 | 11801.85 | 26494.45 | 23065.30 | 30250.38 | 39253.45 | 34505.98 | 44763.17 | 28966.11 | 25426.73 | 32854.62 | 68219.56 | 60186.97 | 77243.82 |
| Turkey | 94741.57 | 85050.17 | 104927.51 | 69055.19 | 61875.02 | 76863.57 | 163796.76 | 148271.96 | 180468.82 | 147131.76 | 131893.53 | 165086.50 | 134284.97 | 119651.66 | 149490.34 | 281416.73 | 252885.72 | 312698.68 |
| United Arab Emirates | 2105.73 | 1814.70 | 2463.22 | 616.99 | 537.33 | 699.96 | 2722.72 | 2358.83 | 3159.00 | 23281.15 | 19836.40 | 27395.87 | 4332.88 | 3756.34 | 5010.56 | 27614.03 | 23611.90 | 32367.94 |
| Yemen | 17266.65 | 15109.16 | 19652.78 | 12419.67 | 10917.92 | 14057.34 | 29686.33 | 26136.94 | 33323.16 | 46293.52 | 41116.40 | 51944.54 | 32806.32 | 29028.82 | 36983.95 | 79099.84 | 70449.37 | 88561.03 |
| Changes* |  | | | | | |  | | | | | |  | | | | | |
| LUI and UUI is the 2·5 to 97·5 centile values of the 95% uncertainty intervals. *: Changes is the total mean values at 2019 compared to 1990. | | | | | | | | | | | | | | | | | | |

Supplementary Table 5.

| Supplementary: Age-standardized DALYs rate of IHD by countries and sex (1990 and 2019) | | | | | | | | | | | | | | | | | | |
| --- | --- | --- | --- | --- | --- | --- | --- | --- | --- | --- | --- | --- | --- | --- | --- | --- | --- | --- |
|  | 1990 | | | | | | | | | 2019 | | | | | | | | |
|  | Male | | | Female | | | Both | | | Male | | | Female | | | Both | | |
|  | Mean | LUI | UUI | Mean | LUI | UUI | Mean | LUI | UUI | Mean | LUI | UUI | Mean | LUI | UUI | Mean | LUI | UUI |
| Global | 4003.52 | 3840.47 | 4147.26 | 2366.31 | 2222.27 | 2491.26 | 3143.28 | 3012.81 | 3257.17 | 2899.51 | 2681.07 | 3117.87 | 1637.86 | 1486.52 | 1769.83 | 2243.54 | 2098.70 | 2385.01 |
| MENA | 7376.15 | 6798.63 | 8043.68 | 5038.41 | 4639.79 | 5470.56 | 6232.35 | 5783.40 | 6705.69 | 4853.00 | 4240.26 | 5611.15 | 3420.28 | 2963.07 | 3899.42 | 4158.94 | 3650.74 | 4751.71 |
| Afghanistan | 10105.85 | 7917.48 | 12721.26 | 8452.68 | 6587.27 | 10783.97 | 9297.15 | 7407.11 | 11625.33 | 7187.98 | 5568.05 | 8740.03 | 6608.19 | 5054.82 | 8563.18 | 6883.50 | 5387.21 | 8475.38 |
| Algeria | 7644.12 | 6225.02 | 9262.21 | 6056.16 | 5088.70 | 7110.64 | 6786.64 | 5676.66 | 8063.19 | 4011.97 | 3107.48 | 5116.66 | 3612.69 | 2970.04 | 4400.45 | 3763.11 | 3049.93 | 4657.02 |
| Bahrain | 9408.42 | 8253.20 | 10609.41 | 6723.46 | 5942.54 | 7557.97 | 8254.04 | 7309.32 | 9242.96 | 2742.79 | 2208.47 | 3370.99 | 2217.58 | 1846.35 | 2665.72 | 2552.30 | 2101.25 | 3090.38 |
| Egypt | 8920.82 | 8076.99 | 10172.53 | 7225.53 | 6511.26 | 8239.16 | 8085.34 | 7486.43 | 8913.91 | 7430.93 | 5596.04 | 9725.48 | 6788.35 | 5140.23 | 8442.55 | 6986.42 | 5361.90 | 8973.94 |
| Iran | 6502.03 | 6020.94 | 7142.42 | 4260.75 | 3841.22 | 4634.40 | 5437.60 | 5060.86 | 5851.02 | 3393.61 | 3160.62 | 3734.03 | 2298.41 | 2100.47 | 2483.96 | 2842.75 | 2657.00 | 3103.12 |
| Iraq | 7538.87 | 6167.14 | 9159.61 | 5042.75 | 4222.49 | 5983.10 | 6281.45 | 5368.85 | 7318.89 | 5941.16 | 4737.96 | 7044.15 | 3644.75 | 3007.38 | 4392.41 | 4775.69 | 3863.59 | 5709.06 |
| Jordan | 4875.82 | 4162.53 | 5710.61 | 3816.06 | 3245.39 | 4429.02 | 4382.81 | 3842.32 | 4982.28 | 2764.59 | 2211.87 | 3442.75 | 1694.17 | 1389.93 | 2057.15 | 2265.22 | 1931.87 | 2687.20 |
| Kuwait | 4449.16 | 4099.90 | 4815.85 | 3163.34 | 2880.56 | 3499.07 | 4013.98 | 3729.80 | 4309.17 | 3155.40 | 2559.13 | 3861.00 | 990.56 | 802.45 | 1202.78 | 2252.19 | 1881.58 | 2687.81 |
| Lebanon | 9031.96 | 7616.27 | 10657.70 | 4849.26 | 4170.67 | 5684.16 | 6914.61 | 5990.59 | 8046.14 | 6370.29 | 4826.03 | 7395.43 | 2923.97 | 1919.43 | 3445.72 | 4491.97 | 3316.33 | 5196.53 |
| Libya | 4221.90 | 3340.06 | 5633.77 | 3370.14 | 2802.49 | 4058.28 | 3831.86 | 3154.32 | 4834.38 | 3875.95 | 2956.86 | 5525.91 | 2958.30 | 2325.96 | 3780.16 | 3428.97 | 2699.30 | 4556.74 |
| Morocco | 7119.33 | 6017.75 | 8452.34 | 5151.69 | 4426.40 | 5991.02 | 6114.72 | 5347.42 | 6928.70 | 5955.15 | 4578.27 | 7153.08 | 4322.55 | 3441.03 | 5233.09 | 5131.83 | 4026.02 | 6088.91 |
| Oman | 10298.85 | 8083.35 | 12722.04 | 7651.27 | 6127.60 | 9425.75 | 9125.88 | 7242.90 | 11207.84 | 5903.44 | 5139.67 | 6794.82 | 4848.08 | 4283.67 | 5477.32 | 5417.45 | 4890.81 | 6023.60 |
| Palestine | 7497.82 | 5992.76 | 9260.87 | 4164.52 | 3353.33 | 5117.59 | 5657.32 | 4531.70 | 6982.59 | 4744.33 | 4117.07 | 5445.22 | 2910.57 | 2528.12 | 3344.46 | 3788.58 | 3300.11 | 4337.98 |
| Qatar | 8025.65 | 6422.56 | 9729.13 | 5900.44 | 4490.33 | 7128.41 | 7161.85 | 5933.31 | 8494.79 | 3336.97 | 2583.50 | 4159.53 | 4426.66 | 3675.97 | 5241.37 | 3597.19 | 2863.86 | 4394.89 |
| Saudi Arabia | 5196.92 | 4026.64 | 6416.05 | 4281.39 | 3470.88 | 5226.91 | 4830.87 | 3863.07 | 5859.23 | 4773.66 | 3932.71 | 5703.80 | 3371.15 | 2731.57 | 4149.63 | 4221.82 | 3508.40 | 5008.79 |
| Sudan | 8980.35 | 6855.16 | 11179.35 | 6173.02 | 4990.63 | 7545.11 | 7625.12 | 6145.22 | 9237.55 | 6073.05 | 4375.87 | 8091.97 | 4409.56 | 3392.85 | 5736.27 | 5290.10 | 4103.35 | 6787.13 |
| Syrian Arab Republic | 9096.19 | 7352.81 | 11076.10 | 6587.20 | 5398.21 | 7866.53 | 7885.82 | 6504.05 | 9453.26 | 7468.63 | 5765.19 | 9771.25 | 5686.87 | 4555.74 | 7156.88 | 6479.76 | 5077.38 | 8364.95 |
| Tunisia | 5277.85 | 4499.96 | 6190.81 | 3350.82 | 2815.39 | 3892.28 | 4324.18 | 3799.93 | 4963.54 | 4305.26 | 3177.36 | 5622.31 | 2465.13 | 1822.86 | 3136.19 | 3357.38 | 2517.60 | 4339.07 |
| Turkey | 6482.19 | 5475.56 | 7474.17 | 3167.74 | 2722.52 | 3663.56 | 4790.09 | 4198.41 | 5381.24 | 2767.50 | 2221.15 | 3386.12 | 1525.92 | 1245.04 | 1867.08 | 2128.38 | 1728.75 | 2583.49 |
| United Arab Emirates | 6035.91 | 4744.60 | 7752.52 | 4366.26 | 3436.29 | 5521.96 | 5526.74 | 4596.73 | 6821.42 | 3719.10 | 2743.02 | 4896.77 | 2322.33 | 1826.13 | 2985.71 | 3370.13 | 2554.97 | 4399.53 |
| Yemen | 9201.74 | 7060.29 | 11911.20 | 5895.01 | 4707.35 | 7335.15 | 7520.41 | 5906.62 | 9540.02 | 6987.02 | 5365.16 | 9301.01 | 4760.37 | 3828.50 | 6181.30 | 5859.78 | 4694.65 | 7645.44 |
| Changes* |  | | | | | |  | | | | | |  | | | | | |
| LUI and UUI is the 2·5 to 97·5 centile values of the 95% uncertainty intervals. *: Changes is the total mean values at 2019 compared to 1990. | | | | | | | | | | | | | | | | | | |

Supplementary Table 6.

| Supplementary: DALYs number of IHD by countries and sex (1990 and 2019) | | | | | | | | | | | | | | | | | | |
| --- | --- | --- | --- | --- | --- | --- | --- | --- | --- | --- | --- | --- | --- | --- | --- | --- | --- | --- |
|  | 1990 | | | | | | | | | 2019 | | | | | | | | |
|  | Male | | | Female | | | Both | | | Male | | | Female | | | Both | | |
|  | Mean | LUI | UUI | Mean | LUI | UUI | Mean | LUI | UUI | Mean | LUI | UUI | Mean | LUI | UUI | Mean | LUI | UUI |
| Global | 72227379.05 | 69305133.98 | 75003874.17 | 48841495.51 | 46134154.20 | 51457887.76 | 121068874.56 | 116357403.14 | 125633899.51 | 110683661.20 | 102235951.50 | 118954617.46 | 71346483.11 | 64769328.51 | 77093702.31 | 182030144.31 | 170206778.29 | 193504630.42 |
| MENA | 6654994.73 | 6133099.09 | 7284506.42 | 4206911.92 | 3889350.64 | 4587268.79 | 10861906.66 | 10042740.60 | 11729607.08 | 11033508.85 | 9511629.20 | 12905429.60 | 6961313.06 | 5988106.81 | 8025461.98 | 17994821.91 | 15580582.01 | 20811862.23 |
| Afghanistan | 378153.56 | 294318.60 | 481148.76 | 299938.88 | 228007.58 | 394139.43 | 678092.44 | 530834.38 | 861085.37 | 512315.27 | 385679.58 | 648699.33 | 471914.89 | 345698.37 | 644141.92 | 984230.16 | 750693.25 | 1275467.35 |
| Algeria | 444341.85 | 354016.08 | 549624.50 | 316591.15 | 258463.28 | 381000.19 | 760933.00 | 620946.80 | 918669.07 | 663469.86 | 506417.16 | 864901.90 | 504142.05 | 406502.40 | 628534.30 | 1167611.91 | 927773.51 | 1464818.68 |
| Bahrain | 10506.22 | 9087.60 | 11964.38 | 4810.63 | 4203.37 | 5465.48 | 15316.85 | 13457.42 | 17353.31 | 16703.99 | 13213.86 | 20788.11 | 6442.62 | 5336.10 | 7877.38 | 23146.62 | 18662.01 | 28406.21 |
| Egypt | 1360377.79 | 1233593.12 | 1590492.13 | 1026511.53 | 924404.13 | 1182166.30 | 2386889.33 | 2209976.21 | 2666487.77 | 2674100.56 | 1985771.88 | 3546774.41 | 1745837.65 | 1283878.45 | 2241391.64 | 4419938.21 | 3313612.83 | 5806683.59 |
| Iran | 901371.81 | 830022.88 | 995658.43 | 491486.75 | 451951.38 | 537892.10 | 1392858.55 | 1303028.27 | 1515544.57 | 1256598.88 | 1175476.60 | 1399982.01 | 768825.50 | 709374.25 | 825789.47 | 2025424.38 | 1898317.78 | 2228260.66 |
| Iraq | 295503.67 | 239920.58 | 362091.40 | 198959.94 | 166286.27 | 237535.02 | 494463.60 | 420840.65 | 578352.90 | 668211.35 | 522276.33 | 819188.33 | 402720.60 | 323038.63 | 496106.59 | 1070931.95 | 845994.89 | 1307890.40 |
| Jordan | 36510.40 | 30693.00 | 43226.60 | 22597.82 | 18982.05 | 26497.00 | 59108.22 | 51110.70 | 67884.69 | 102393.02 | 81331.64 | 127820.51 | 45087.60 | 36552.09 | 55577.37 | 147480.63 | 124435.73 | 177497.89 |
| Kuwait | 21903.79 | 20283.30 | 23746.68 | 7303.53 | 6712.98 | 7973.94 | 29207.32 | 27368.96 | 31545.89 | 61559.44 | 49811.00 | 75559.38 | 10117.47 | 8221.93 | 12460.37 | 71676.91 | 59488.76 | 86115.33 |
| Lebanon | 102677.29 | 86116.13 | 122333.75 | 52717.93 | 45337.48 | 62094.30 | 155395.22 | 133566.05 | 182156.98 | 150141.04 | 113571.41 | 174572.93 | 83230.57 | 54696.49 | 98006.70 | 233371.61 | 171955.13 | 270077.00 |
| Libya | 45364.92 | 35615.37 | 60774.40 | 30423.64 | 25126.98 | 36901.57 | 75788.56 | 62330.80 | 96554.25 | 107834.17 | 81868.73 | 157427.86 | 75324.49 | 58776.57 | 97323.24 | 183158.66 | 144661.89 | 247389.16 |
| Morocco | 488272.53 | 409855.50 | 581743.78 | 349982.85 | 299002.25 | 411835.89 | 838255.38 | 727881.53 | 958415.08 | 900264.79 | 670636.06 | 1098920.91 | 650757.78 | 506419.43 | 807142.47 | 1551022.57 | 1191648.77 | 1885658.24 |
| Oman | 39355.79 | 29853.29 | 50405.49 | 21321.32 | 16902.18 | 26969.14 | 60677.11 | 47072.58 | 76186.10 | 53786.13 | 44802.42 | 64884.24 | 29427.31 | 25667.62 | 33642.39 | 83213.45 | 72529.34 | 95689.93 |
| Palestine | 29551.74 | 23395.50 | 36912.60 | 19026.42 | 15211.32 | 23459.59 | 48578.16 | 38488.92 | 60687.19 | 54225.40 | 46742.24 | 62336.08 | 32479.12 | 28072.57 | 37477.08 | 86704.52 | 75038.22 | 99657.10 |
| Qatar | 5783.18 | 4642.72 | 7154.03 | 1921.91 | 1544.64 | 2329.61 | 7705.09 | 6321.19 | 9277.85 | 19149.51 | 14229.76 | 25372.20 | 5070.88 | 3960.22 | 6300.88 | 24220.39 | 18354.90 | 31467.30 |
| Saudi Arabia | 188962.64 | 142636.22 | 241392.37 | 105240.45 | 83901.07 | 130072.21 | 294203.09 | 227774.45 | 365465.29 | 619753.96 | 488266.12 | 784938.36 | 263805.50 | 208854.08 | 330254.79 | 883559.46 | 702672.55 | 1099539.69 |
| Sudan | 453428.64 | 343821.28 | 572256.03 | 281646.76 | 224019.41 | 350856.99 | 735075.40 | 583679.35 | 900700.76 | 638180.27 | 452512.99 | 870822.00 | 381313.72 | 279311.97 | 516989.44 | 1019493.99 | 771848.59 | 1347945.11 |
| Syrian Arab Republic | 275549.99 | 221647.23 | 336788.15 | 172066.54 | 138617.05 | 208209.33 | 447616.53 | 365626.51 | 536716.20 | 472480.82 | 358271.21 | 627570.11 | 292538.23 | 226855.16 | 382260.25 | 765019.05 | 586984.15 | 1001671.78 |
| Tunisia | 132922.15 | 112259.96 | 157670.81 | 75964.74 | 63920.37 | 89099.27 | 208886.90 | 182152.11 | 241801.51 | 255876.15 | 186980.46 | 338184.94 | 152811.67 | 112703.29 | 195400.82 | 408687.82 | 304295.77 | 531699.78 |
| Turkey | 1182866.96 | 994915.72 | 1379394.17 | 571100.91 | 493149.41 | 666965.90 | 1753967.88 | 1529781.33 | 1979197.42 | 1149727.84 | 921687.44 | 1415156.55 | 697315.93 | 568569.47 | 854374.22 | 1847043.77 | 1491706.84 | 2245824.30 |
| United Arab Emirates | 21724.69 | 16339.80 | 29468.94 | 4968.35 | 3797.07 | 6361.66 | 26693.04 | 20815.69 | 34968.95 | 157127.88 | 109398.34 | 222668.86 | 17264.46 | 12980.21 | 22629.86 | 174392.34 | 124283.75 | 243499.79 |
| Yemen | 235389.09 | 174715.62 | 311365.57 | 149500.37 | 115911.18 | 189387.01 | 384889.46 | 293167.90 | 495330.45 | 488398.64 | 365692.52 | 662522.78 | 317812.44 | 243901.96 | 423539.29 | 806211.08 | 620074.29 | 1081163.14 |
| Changes* |  | | | | | |  | | | | | |  | | | | | |
| LUI and UUI is the 2·5 to 97·5 centile values of the 95% uncertainty intervals. *: Changes is the total mean values at 2019 compared to 1990. | | | | | | | | | | | | | | | | | | |

Supplementary Table 7.

| Supplementary: Age-standardized death rate of IHD by countries and sex (1990 and 2019) | | | | | | | | | | | | | | | | | | |
| --- | --- | --- | --- | --- | --- | --- | --- | --- | --- | --- | --- | --- | --- | --- | --- | --- | --- | --- |
|  | 1990 | | | | | | | | | 2019 | | | | | | | | |
|  | Male | | | Female | | | Both | | | Male | | | Female | | | Both | | |
|  | Mean | LUI | UUI | Mean | LUI | UUI | Mean | LUI | UUI | Mean | LUI | UUI | Mean | LUI | UUI | Mean | LUI | UUI |
| Global | 205.24 | 194.29 | 213.05 | 141.73 | 130.00 | 149.72 | 170.45 | 159.61 | 176.94 | 144.60 | 132.87 | 154.96 | 95.07 | 83.91 | 103.11 | 117.95 | 107.83 | 125.92 |
| MENA | 349.11 | 318.94 | 378.15 | 268.56 | 244.28 | 292.94 | 309.32 | 284.33 | 332.13 | 241.73 | 213.77 | 274.66 | 195.16 | 169.43 | 218.80 | 219.01 | 194.15 | 246.75 |
| Afghanistan | 439.29 | 351.44 | 537.61 | 379.11 | 310.56 | 458.72 | 410.08 | 337.36 | 493.65 | 326.72 | 254.48 | 390.43 | 316.39 | 246.60 | 396.67 | 320.87 | 253.97 | 385.29 |
| Algeria | 414.77 | 346.88 | 492.17 | 387.47 | 332.86 | 448.53 | 395.48 | 337.77 | 457.93 | 230.62 | 184.60 | 283.36 | 257.87 | 214.26 | 308.30 | 237.25 | 197.13 | 282.69 |
| Bahrain | 488.78 | 434.14 | 544.59 | 388.09 | 342.49 | 430.03 | 442.46 | 394.45 | 490.13 | 159.29 | 128.99 | 196.33 | 147.31 | 122.31 | 174.42 | 155.09 | 129.29 | 186.67 |
| Egypt | 425.56 | 382.87 | 470.54 | 395.62 | 355.76 | 448.82 | 410.76 | 377.90 | 449.55 | 349.62 | 269.48 | 445.75 | 404.90 | 314.20 | 486.81 | 359.27 | 281.82 | 447.03 |
| Iran | 322.07 | 295.45 | 352.94 | 245.55 | 213.62 | 269.07 | 285.68 | 260.83 | 307.09 | 177.18 | 161.53 | 192.51 | 150.93 | 133.83 | 164.00 | 163.56 | 148.98 | 176.17 |
| Iraq | 356.78 | 293.20 | 422.99 | 267.16 | 223.53 | 313.13 | 311.21 | 265.52 | 357.49 | 304.61 | 249.20 | 349.64 | 209.49 | 176.41 | 245.40 | 255.40 | 214.11 | 292.61 |
| Jordan | 226.57 | 193.21 | 264.01 | 222.32 | 190.25 | 256.56 | 225.39 | 197.81 | 254.57 | 135.08 | 108.11 | 167.19 | 107.79 | 87.86 | 129.37 | 121.92 | 103.15 | 144.05 |
| Kuwait | 202.69 | 182.12 | 219.44 | 173.91 | 154.21 | 192.53 | 194.57 | 175.56 | 208.87 | 142.92 | 116.01 | 174.10 | 58.58 | 46.35 | 71.02 | 108.53 | 90.73 | 129.20 |
| Lebanon | 432.53 | 367.55 | 507.12 | 276.60 | 234.78 | 323.64 | 352.64 | 307.84 | 405.86 | 322.56 | 238.37 | 370.20 | 174.97 | 113.83 | 202.57 | 241.23 | 174.11 | 277.12 |
| Libya | 204.65 | 158.62 | 271.43 | 173.28 | 139.70 | 209.39 | 189.88 | 153.37 | 236.74 | 188.13 | 142.89 | 262.59 | 153.84 | 120.45 | 195.53 | 171.30 | 135.45 | 226.05 |
| Morocco | 345.77 | 291.42 | 407.81 | 270.17 | 231.27 | 310.75 | 306.23 | 264.97 | 346.06 | 314.01 | 247.68 | 366.75 | 244.16 | 199.36 | 287.72 | 278.54 | 224.66 | 321.41 |
| Oman | 524.28 | 422.98 | 634.68 | 417.21 | 339.43 | 501.20 | 470.15 | 382.97 | 562.72 | 361.87 | 317.20 | 414.69 | 299.99 | 264.68 | 338.67 | 329.85 | 296.03 | 364.09 |
| Palestine | 366.63 | 298.05 | 443.22 | 229.77 | 188.80 | 279.18 | 289.88 | 238.10 | 349.39 | 253.02 | 220.01 | 288.67 | 173.50 | 149.85 | 199.04 | 207.16 | 180.07 | 236.15 |
| Qatar | 458.50 | 357.61 | 547.54 | 348.47 | 260.76 | 418.46 | 405.98 | 334.40 | 473.78 | 230.47 | 181.69 | 283.23 | 332.93 | 281.55 | 387.27 | 252.99 | 205.75 | 305.43 |
| Saudi Arabia | 255.34 | 202.38 | 306.49 | 221.97 | 181.06 | 266.68 | 241.31 | 196.57 | 285.82 | 225.63 | 191.21 | 261.37 | 174.91 | 142.02 | 213.10 | 205.60 | 172.88 | 238.97 |
| Sudan | 412.63 | 318.49 | 506.18 | 307.85 | 249.57 | 371.99 | 361.78 | 292.57 | 428.25 | 298.45 | 220.32 | 385.84 | 240.43 | 190.58 | 301.85 | 271.47 | 214.92 | 338.38 |
| Syrian Arab Republic | 414.67 | 339.49 | 496.65 | 359.14 | 297.07 | 419.32 | 387.72 | 323.52 | 455.31 | 376.36 | 295.18 | 477.86 | 369.60 | 305.32 | 446.55 | 359.72 | 288.25 | 449.75 |
| Tunisia | 281.79 | 242.57 | 328.65 | 197.18 | 163.61 | 228.43 | 238.79 | 208.11 | 273.08 | 237.39 | 179.63 | 302.50 | 154.27 | 114.00 | 194.23 | 193.45 | 146.86 | 244.04 |
| Turkey | 289.40 | 246.36 | 331.05 | 174.73 | 147.62 | 203.12 | 229.86 | 199.98 | 255.18 | 143.97 | 116.73 | 174.77 | 100.11 | 80.33 | 123.61 | 120.96 | 97.99 | 147.20 |
| United Arab Emirates | 308.48 | 249.54 | 388.62 | 270.69 | 214.76 | 342.94 | 297.53 | 251.60 | 366.43 | 187.24 | 140.05 | 240.61 | 143.14 | 113.90 | 182.12 | 175.40 | 134.75 | 223.39 |
| Yemen | 420.89 | 330.75 | 529.68 | 296.82 | 242.82 | 360.20 | 353.65 | 286.77 | 434.72 | 336.79 | 265.10 | 433.95 | 254.67 | 208.47 | 321.02 | 294.76 | 243.73 | 374.52 |
| Changes* |  | | | | | |  | | | | | |  | | | | | |
| LUI and UUI is the 2·5 to 97·5 centile values of the 95% uncertainty intervals. *: Changes is the total mean values at 2019 compared to 1990. | | | | | | | | | | | | | | | | | | |

Supplementary Table 8.

| Supplementary: Death number of IHD by countries and sex (1990 and 2019) | | | | | | | | | | | | | | | | | | |
| --- | --- | --- | --- | --- | --- | --- | --- | --- | --- | --- | --- | --- | --- | --- | --- | --- | --- | --- |
|  | 1990 | | | | | | | | | 2019 | | | | | | | | |
|  | Male | | | Female | | | Both | | | Male | | | Female | | | Both | | |
|  | Mean | LUI | UUI | Mean | LUI | UUI | Mean | LUI | UUI | Mean | LUI | UUI | Mean | LUI | UUI | Mean | LUI | UUI |
| Global | 3022459.34 | 2895626.91 | 3133481.84 | 2673430.76 | 2478701.46 | 2815919.15 | 5695890.11 | 5405191.11 | 5895398.09 | 4968251.35 | 4591342.95 | 5344570.79 | 4169539.80 | 3680480.57 | 4521720.49 | 9137791.14 | 8395682.48 | 9743550.13 |
| MENA | 257771.59 | 237189.09 | 279881.50 | 186918.78 | 172234.22 | 202900.25 | 444690.36 | 411687.84 | 478245.11 | 460159.80 | 404384.24 | 528542.92 | 339324.59 | 295245.06 | 381948.08 | 799484.40 | 706348.60 | 909786.81 |
| Afghanistan | 14724.94 | 11527.95 | 18322.67 | 11385.01 | 9011.15 | 14153.43 | 26109.94 | 21043.41 | 31907.99 | 17534.74 | 13540.91 | 21525.29 | 17093.05 | 12944.13 | 22053.01 | 34627.79 | 26988.79 | 42664.91 |
| Algeria | 18224.50 | 14764.79 | 22131.42 | 13834.78 | 11530.17 | 16373.04 | 32059.28 | 26644.69 | 38231.55 | 31313.90 | 24530.78 | 39295.31 | 27378.17 | 22571.61 | 33351.94 | 58692.07 | 47672.64 | 71562.90 |
| Bahrain | 355.15 | 309.24 | 402.43 | 205.56 | 181.34 | 232.38 | 560.71 | 494.63 | 630.44 | 566.27 | 444.49 | 706.45 | 297.09 | 244.80 | 362.98 | 863.35 | 697.71 | 1059.73 |
| Egypt | 52229.77 | 47161.84 | 59091.76 | 44518.55 | 39919.48 | 50846.88 | 96748.31 | 89582.94 | 106510.45 | 105591.55 | 79862.89 | 138053.43 | 76293.02 | 57148.83 | 95631.97 | 181884.57 | 138958.56 | 233632.32 |
| Iran | 34038.38 | 31467.83 | 37505.37 | 21629.94 | 19335.35 | 23582.05 | 55668.31 | 51802.44 | 60039.05 | 58319.99 | 53654.01 | 63322.98 | 44478.79 | 39745.43 | 48195.09 | 102798.78 | 94454.77 | 111215.26 |
| Iraq | 12084.26 | 9913.77 | 14543.50 | 9471.92 | 7906.09 | 11160.95 | 21556.17 | 18421.58 | 24920.68 | 27493.41 | 21930.45 | 32506.99 | 19354.53 | 16137.42 | 23187.91 | 46847.94 | 38262.60 | 55510.86 |
| Jordan | 1318.03 | 1120.79 | 1543.40 | 1049.10 | 892.47 | 1214.45 | 2367.12 | 2075.53 | 2691.71 | 3882.66 | 3097.65 | 4871.47 | 2228.30 | 1806.79 | 2709.44 | 6110.96 | 5195.84 | 7285.93 |
| Kuwait | 692.07 | 638.82 | 749.01 | 328.43 | 296.07 | 361.74 | 1020.49 | 947.60 | 1097.79 | 2132.47 | 1724.84 | 2606.20 | 466.72 | 372.31 | 572.27 | 2599.19 | 2165.34 | 3105.42 |
| Lebanon | 4158.75 | 3509.11 | 4913.58 | 2607.66 | 2229.90 | 3054.81 | 6766.41 | 5872.66 | 7839.10 | 7340.68 | 5433.11 | 8438.89 | 4910.74 | 3182.27 | 5683.69 | 12251.42 | 8865.73 | 14092.29 |
| Libya | 1819.80 | 1424.13 | 2432.61 | 1384.09 | 1128.99 | 1666.01 | 3203.89 | 2593.87 | 4005.78 | 4399.55 | 3340.98 | 6265.17 | 3427.26 | 2695.32 | 4343.70 | 7826.81 | 6177.05 | 10362.77 |
| Morocco | 19669.36 | 16647.13 | 23208.04 | 15626.65 | 13459.85 | 17999.09 | 35296.02 | 31030.94 | 39850.19 | 40444.14 | 31391.03 | 47748.63 | 31567.39 | 25359.74 | 37569.67 | 72011.52 | 56906.33 | 84500.53 |
| Oman | 1398.93 | 1075.27 | 1757.15 | 965.66 | 774.60 | 1182.22 | 2364.59 | 1858.62 | 2911.35 | 2037.49 | 1739.62 | 2408.16 | 1374.16 | 1210.49 | 1551.92 | 3411.65 | 3045.72 | 3828.43 |
| Palestine | 1267.87 | 1021.72 | 1549.13 | 948.22 | 773.63 | 1159.72 | 2216.09 | 1797.63 | 2715.06 | 2141.87 | 1859.32 | 2469.50 | 1667.95 | 1443.05 | 1919.86 | 3809.82 | 3325.69 | 4366.03 |
| Qatar | 189.85 | 152.21 | 234.54 | 83.79 | 65.13 | 101.02 | 273.64 | 225.82 | 326.83 | 620.13 | 459.27 | 817.52 | 209.88 | 165.79 | 259.44 | 830.01 | 630.61 | 1067.60 |
| Saudi Arabia | 7159.29 | 5533.84 | 8871.46 | 4598.08 | 3726.73 | 5548.56 | 11757.38 | 9412.71 | 14193.53 | 20273.75 | 16270.32 | 24883.52 | 9415.31 | 7564.86 | 11697.62 | 29689.06 | 24089.22 | 36175.78 |
| Sudan | 17639.73 | 13516.58 | 21846.96 | 11743.79 | 9485.81 | 14285.05 | 29383.52 | 23723.49 | 35303.36 | 26100.16 | 19124.95 | 34245.26 | 17087.20 | 13250.78 | 22186.21 | 43187.36 | 33612.67 | 54936.11 |
| Syrian Arab Republic | 10525.70 | 8558.07 | 12751.66 | 7496.68 | 6170.78 | 8824.85 | 18022.39 | 14948.43 | 21346.35 | 19843.11 | 15266.34 | 25955.72 | 13698.43 | 10890.84 | 17325.02 | 33541.55 | 26238.92 | 43170.04 |
| Tunisia | 5813.43 | 4941.13 | 6818.58 | 3648.12 | 3062.52 | 4235.20 | 9461.55 | 8283.03 | 10856.19 | 12479.62 | 9360.57 | 16158.42 | 8977.41 | 6597.79 | 11364.56 | 21457.03 | 16189.46 | 27280.55 |
| Turkey | 45436.06 | 38390.02 | 52156.28 | 28760.52 | 24366.00 | 33449.00 | 74196.58 | 64620.25 | 82836.72 | 54253.82 | 43622.82 | 66073.29 | 44792.56 | 35977.83 | 55356.70 | 99046.38 | 80454.39 | 120867.09 |
| United Arab Emirates | 636.44 | 488.30 | 848.33 | 201.43 | 156.43 | 259.16 | 837.88 | 666.72 | 1072.76 | 4291.47 | 2994.54 | 6020.63 | 588.66 | 446.69 | 770.57 | 4880.13 | 3503.69 | 6754.69 |
| Yemen | 8215.91 | 6252.52 | 10727.83 | 6305.10 | 5092.35 | 7792.25 | 14521.01 | 11428.58 | 18316.30 | 18631.51 | 14340.66 | 24650.82 | 13673.24 | 11009.28 | 17616.03 | 32304.75 | 26019.79 | 42118.22 |
| Changes* |  | | | | | |  | | | | | |  | | | | | |
| LUI and UUI is the 2·5 to 97·5 centile values of the 95% uncertainty intervals. *: Changes is the total mean values at 2019 compared to 1990. | | | | | | | | | | | | | | | | | | |
